# Immune response to the hepatitis B antigen in the RTS,S/AS01 malaria vaccine, and co-administration with pneumococcal conjugate and rotavirus vaccines in African children: A randomized controlled trial

**DOI:** 10.1080/21645515.2018.1442996

**Published:** 2018-04-13

**Authors:** Innocent Valéa, Samuel Adjei, Effua Usuf, Ousmane Traore, Daniel Ansong, Halidou Tinto, Harry Owusu Boateng, Amanda Leach, Athanase Mwinessobaonfou Some, Patrick Buabeng, Johan Vekemans, Louis Arnaud Nana, Amos Kotey, Pascale Vandoolaeghe, Florence Ouedraogo, David Sambian, Marc Lievens, Marc Christian Tahita, Theresa Rettig, Erik Jongert, Palpouguini Lompo, Ali Idriss, Dorota Borys, Sayouba Ouedraogo, Frank Prempeh, Md Ahsan Habib, Lode Schuerman, Hermann Sorgho, Tsiri Agbenyega

**Affiliations:** a Institut de Recherche en Sciences de la Santé, Nanoro, Burkina Faso; b School of Medical Sciences, KNUST, Kumasi (Agogo), Ghana; c GSK, Wavre, Belgium

**Keywords:** co-administration, hepatitis B, immunogenicity, malaria, plasmodium falciparum, pneumococcal conjugate vaccine, RTS,S, vaccine

## Abstract

The RTS,S/AS01 malaria vaccine (*Mosquirix*) reduces the incidence of *Plasmodium falciparum* malaria and is intended for routine administration to infants in Sub-Saharan Africa. We evaluated the immunogenicity and safety of 10-valent pneumococcal non-typeable *Haemophilus influenzae* protein D conjugate vaccine (PHiD-CV; *Synflorix*) and human rotavirus vaccine (HRV; *Rotarix*) when co-administered with RTS,S/AS01 (www.clinicaltrials.gov NCT01345240) in African infants. 705 healthy infants aged 8–12 weeks were randomized to receive three doses of either RTS,S/AS01 or licensed hepatitis B (HBV; *Engerix B*) vaccine (control) co-administered with diphtheria-tetanus-acellular pertussis-*Haemophilus influenzae* type-b-conjugate vaccine (DTaP/Hib) and trivalent oral poliovirus vaccine at 8–12-16 weeks of age, because DTaP/Hib was not indicated before 8 weeks of age. The vaccination schedule can still be considered broadly applicable because it was within the age range recommended for EPI vaccination. PHiD-CV or HRV were either administered together with the study vaccines, or after a 2-week interval. Booster doses of PHiD-CV and DTaP/Hib were administered at age 18 months.

Non-inferiority of anti-HBV surface antigen antibody seroprotection rates following co-administration with RTS,S/AS01 was demonstrated compared to the control group (primary objective). Pre-specified non-inferiority criteria were reached for PHiD-CV (for 9/10 vaccine serotypes), HRV, and aP antigens co-administered with RTS,S/AS01 as compared to HBV co-administration (secondary objectives). RTS,S/AS01 induced a response to circumsporozoite protein in all groups. Pain and low grade fever were reported more frequently in the PHiD-CV group co-administered with RTS,S/AS01 than PHiD-CV co-administered with HBV. No serious adverse events were considered to be vaccine-related. RTS,S/AS01 co-administered with pediatric vaccines had an acceptable safety profile. Immune responses to RTS,S/AS01 and to co-administered PHiD-CV, pertussis antigens and HRV were satisfactory.

## Introduction

The World Health Organization (WHO) estimated that in 2015 there were 214 million cases of malaria and 428,000 deaths; the majority of which (88%) occurred in Africa.^1^ Around 71% of malaria deaths in 2015 were in children under 5 years of age. While improvements in prevention (increasing use of insecticide-treated bed nets and chemoprevention), diagnosis and treatment have contributed to a 37% decline in the incidence of malaria between 2000 and 2015, malaria remains a leading cause of childhood death in Africa.^1^ An effective vaccine delivered through the existing Expanded Programme on Immunisation (EPI) delivery platform, could play an important additional role in malaria control.

The RTS,S/AS01 malaria vaccine (*Mosquirix*) contains the RTS,S hybrid antigen; a portion of the *Plasmodium falciparum* circumsporozoite (CS) protein fused to hepatitis B virus surface antigen (HBsAg). The fused antigen is expressed together with unfused HBsAg in yeast. RTS,S/AS01 induces antibody responses to the CS protein and to HBsAg. Vaccine efficacy after four RTS,S/AS01 doses administered to 5–17 months-old children was 43.9% (95% confidence interval [CI] 39.7; 47.8) against clinical malaria over 32 months of follow-up, and 27.8% (95% CI 21.7; 33.4) in 6–12 weeks old infants.^2^

Co-administration of RTS,S with combined diphtheria-tetanus-whole-cell pertussis-hepatitis B-*Haemophilus influenzae* type b vaccine (DTwP-HBV/Hib) and trivalent oral poliovirus vaccine (tOPV) in infants aged 6–10 weeks at the time of the first dose, showed acceptable safety and immunogenicity of the co-administered antigens.^3^

Here we report the results of a Phase III open randomized study (www.clinicaltrials.gov NCT01345240) designed to investigate whether primary vaccination with RTS,S/AS01 was as immunogenic as licensed HBV (primary objective). Secondary study objectives were the assessment of the immunogenicity and safety of three doses of RTS,S/AS01 compared to HBV when co-administered with pediatric vaccines to infants commencing at 8–12 weeks of age, including pneumococcal conjugate vaccine (PCV) and rotavirus vaccine, recommended within the existing EPI schedule in African countries. In the absence of an available DTwP vaccine that did not contain HBsAg, we used an acellular pertussis (aP)-containing vaccine (DTaP/Hib; *Infanrix* Hib) in this study.

The study design is provided in Supplement Fig. 1. There were five study groups. Infants received three doses of RTS,S/AS01 (group R) or HBV control vaccine (*Engerix B*; group C) at 8, 12 and 16 weeks of age, co-administered with DTaP/Hib (*Infanrix*Hib) and tOPV (*Polio Sabin*) (Table 1). In the groups R1 and C1, 10-valent pneumococcal non-typeable *Haemophilus influenzae* protein D conjugate vaccine (PHiD-CV, *Synflorix*) was administered together with the study vaccines, respectively RTS,S/AS01 or HBV, and in the groups R2 and C2, human rotavirus vaccine (HRV, *Rotarix*) was administered together with the study vaccines, respectively RTS,S/AS01 and HBV, with staggered administration of the other vaccine after a 2-week interval (Table 1). In the group R3, PHiD-CV and HRV were both co-administered two weeks after the RTS,S/AS01 dose. In this report we provide immunogenicity and safety data from post-primary vaccination until study month 26, approximately 2 years after the third dose of RTS,S/AS01 or HBV vaccines.

**Figure 1.**
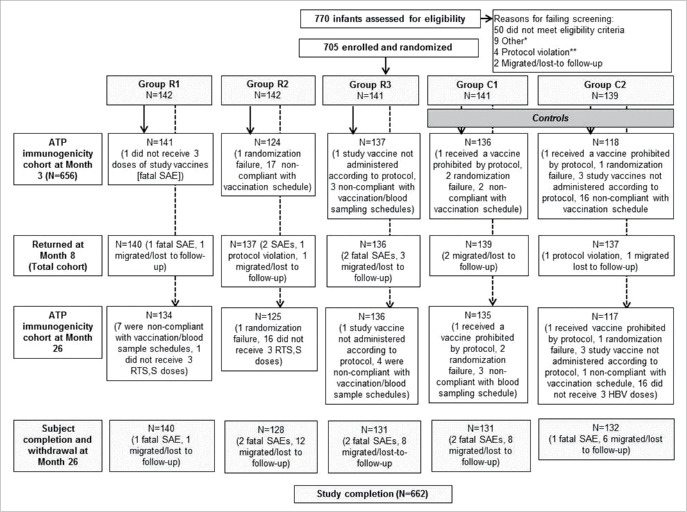
Study flow per co-administration vaccination regimen from week 0 until month 26.Group R1 received RTS,S/AS01 + (DTaP/Hib + tOPV + PHiD-CV), and HRV 2 weeks later, Group R2 received RTS,S/AS01 + (DTaP/Hib + tOPV + HRV), and PHiD-CV 2 weeks later, Group R3 received RTS,S/AS01 + (DTaP/Hib + tOPV), and (PHiD-CV + HRV) 2 weeks later, Group C1 received HBV + (DTaP/Hib + tOPV + PHiD-CV), and HRV 2 weeks later, Group C2 received HBV + (DTaP/Hib + tOPV + HRV), and PHiD-CV 2 weeks later, * Other: five consent withdrawal, two recruitment target reached in SBIR, one down syndrome, and one end of inclusion (recruitment was completed), ** Protocol violation: three screening expired and one child received recommended vaccines before enrolment, DTaP/Hib = diphtheria-tetanus-acellular-pertussis-Haemophilus influenzae type-b-conjugate vaccine, tOPV = trivalent oral poliovirus vaccine, PHiD-CV = 10-valent pneumococcal non-typeable Haemophilus influenzae protein D conjugate vaccine, HRV = human rotavirus vaccine.

**Table 1.** Treatment groups.

		Treatment	Pooled groups	Primary vaccination schedule			Booster	
Name of the group		Group	(primary objective)	Co-administered vaccines	Staggered vaccination^*^	N	18 months of age	N
R	R1	RTS,S/AS01 + PHiD-CV	Pooled RTS,S/AS01 groups (N = 425)	RTS,S/AS01 + DTaP/Hib + tOPV + PHiD-CV	HRV	142	DTaP/Hib + PHiD-CV	425
	R2	RTS,S/AS01 + HRV		RTS,S/AS01 + DTaP/Hib + tOPV + HRV	PHiD-CV	142		
	R3	RTS,S/AS01 alone		RTS,S/AS01 + DTaP/Hib + tOPV	PHiD-CV + HRV	141		
C	C1	HBV + PHiD-CV	Pooled HBV groups (N = 280)	HBV + DTaP/Hib + tOPV + PHiD-CV	HRV	141	DTaP/Hib + PHiD-CV	280
	C2	HBV + HRV		HBV + DTaP/Hib + tOPV + HRV	PHiD-CV	139		

N = number of participants in the Total Vaccinated cohort. DTaP/Hib = *Infanrix* Hib, tOPV = trivalent oral polio vaccine, HRV = human rotavirus vaccine (*Rotarix*), PHiD-CV = 10-valent pneumococcal conjugate vaccine (*Synflorix*), HBV = hepatitis B vaccine (*Engerix* B).

* Staggered administration of the indicated vaccine i.e., given 2 weeks after administration of the other vaccines.

The post-primary results of this study were included in the dossier submitted to the European Medicines Agency (EMA), which granted positive scientific opinion in July 2015 for the prevention of *P. falciparum* malaria and, based on this trial, HBV infection in infants and young children.^4^ The EMA noted that the co-administration of RTS,S/AS01 with PCVs increases the risk of fever within 7 days post-vaccination, and that concomitant administration of rotavirus vaccine and PCV with RTS,S/AS01 may reduce the antibody response to CS. The impact of this observation on the level of protection induced by RTS,S/AS01 is currently unknown. In October 2015, the WHO recommended evaluation of RTS,S/AS01 as a 4-dose schedule for 5–17 months old children in pilot programs prior to its wider use.^5^ RTS,S/AS01 was not recommended for use in infants because of lower efficacy observed in this age group in Phase III. In 2017 the WHO announced that beginning in 2018, RTS,S/AS01 will be made available in selected areas of Ghana, Kenya, and Malawi as part of the Malaria Vaccine Pilot Implementation Programme.^6^

## Results

### Study participants

The first participant was enrolled on 17 October 2011 and the data lock for the immunogenicity follow-up was 22 September 2015. From a total of 770 infants assessed for eligibility, 705 were randomized and vaccinated and 656 contributed to the according to protocol analysis of immunogenicity at month 3 (Fig. 1). The main reason for elimination from the immunogenicity analysis was non-compliance with the vaccination schedule; this primarily affected the groups that received co-administered HRV as there were children at one study site who received the first dose of HRV at visit 2 instead of visit 4. There were 662 participants at the month 26 visit. The reasons for drop out were migration from the study area/lost-to follow-up (n = 35) and death (n = 8). No deaths were considered by the investigator to be related to vaccination (details of fatal serious adverse events [SAEs] are provided in Supplement Table 1).

Participants in the five treatment groups were 8.3–8.4 weeks of age at the time of the first dose (range 8–12 weeks), and 41.5%-57.4% of children in each treatment group were female (Supplement Table 2).

**Table 2.** Results of the inferential analyses for HBV, PHiD-CV, HRV and pertussis vaccine antigens administered as three priming doses during infancy (according to protocol immunogenicity cohort).

Objective	Endpoint	Criteria	Antigen	Value	(95% CI)	Criterion met?
Primary objective						
Non-inferiority group R vs group C	Anti-HBs ≥10mIU/ml	UL of 2-sided 95% CI for difference (HBV *minus* RTS,S/AS01) is <5%	HBsAg	−3.95	(−7.12; −2.16)	Yes
Secondary objectives						
Non-inferiority of group C1 over group R1	IgG GMC ratio	UL of 2-sided 95% CI for ratio is <2 for each pneumococcal vaccine serotype	1	1.15	(0.95; 1.39)	Yes
			4	1.20	(0.97; 1.48)	Yes
			5	1.27	(1.06; 1.52)	Yes
			6B	1.17	(0.83; 1.65)	Yes
			7F	1.12	(0.94; 1.33)	Yes
			9V	1.32	(1.08; 1.63)	Yes
			14	0.99	(0.77; 1.27)	Yes
			18C	1.81	(1.38; 2.38)	No
			19F	1.21	(0.89; 1.65)	Yes
			23F	1.12	(0.81; 1.55)	Yes
Non-inferiority of group C2 over group R2	IgA GMC ratio	UL of 2-sided 95% CI for ratio is <2	HRV	1.11	(0.76; 1.61)	Yes
Non-inferiority group R over group C	GMC ratio	UL of 2-sided 95% CI for ratio is <2	PT	1.08	(0.97; 1.20)	Yes
			FHA	1.08	(0.97; 1.21)	Yes
			PRN	1.10	(0.98; 1.22)	Yes

95% CI – 95% confidence interval; UL– upper limit of the 95% CI; GMC – geometric mean antibody concentration, RTS,S/AS01 –malaria vaccine, HBsAg – hepatitis B surface antigen, HRV – human rotavirus vaccine, PT – pertussis toxoid, FHA – filamentous haemagglutinin, PRN – pertactin, PHiD-CV – 10-valent pneumococcal non-typeable *Haemophilus influenzae* protein D conjugate vaccine.

### Immune response to RTS,S/AS01

#### RTS,S hepatitis B antigen

The primary study objective, to demonstrate the non-inferiority of RTS,S/AS01 to HBV vaccine in terms of anti-HBs antibody seroprotection rates one month post-dose 3 on the three pooled groups that received RTS,S/AS01 (Group R) compared to the pooled groups that received HBV (Group C), was reached (Table 2). One month post-dose 3, all participants in the group R and 96.0% in the group C had anti-HBs concentrations ≥10mIU/ml, with geometric mean antibody concentrations (GMCs) of 6,412.7 mIU/ml and 377.4 mIU/ml respectively (Table 3).

**Table 3.** Anti-HBs seroprotection rates and geometric mean antibody titers one month post-dose 3 (ATP immunogenicity cohort).

			≥ 6.2 mIU/ml			≥ 10 mIU/ml			GMT	
Group	Timing	N	n	%	(95% CI)	n	%	(95% CI)	value	(95% CI)
R	Screening	398	83	20.9	(17.0; 25.2)	63	15.8	(12.4; 19.8)	5.0	(4.5; 5.7)
	Post-III	397	397	100	(99.1; 100)	397	100	(99.1; 100)	6412.7	(5732.9; 7173.0)
C	Screening	251	57	22.7	(17.7; 28.4)	45	17.9	(13.4; 23.2)	5.4	(4.7; 6.4)
	Post-III	253	246	97.2	(94.4; 98.9)	243	96.0	(92.9; 98.1)	377.4	(310.6; 458.7)

Group R = All study groups that received RTS,S/AS01 vaccine.

Group C = All study groups that received HBV vaccine.

N = number of subjects with available results.

GMT = geometric mean antibody titer calculated on all subjects.

n/% = number/percentage of subjects with titer ≥ specified value.

95% CI = 95% confidence interval.

Screening = Pre-vaccination, Post III = one month post-dose 3.

ATP = according to protocol.

#### RTS,S circumsporozoite protein antigen

One month after three RTS,S/AS01 doses, 99.3% to 100% of participants were seropositive for anti-CS antibodies (Table 4). The post-dose 3 anti-CS antibody GMC was 205.5 EU/ml in the group R3 and 142.2 EU/ml in the group R1 (GMC ratio 1.45, 95% CI 1.10; 1.90).

**Table 4.** Anti-CS antibody seropositivity rates and geometric mean concentrations (GMC) one month post-dose 3 (adapted^*^ according to Protocol immunogenicity cohort).

			≥ 0.5 EU/ml			GMC	
Group	Timing	N	n	%	(95% CI)	value	(95% CI)
R1	Screening	141	91	64.5	(56.0; 72.4)	0.7	(0.6; 0.9)
	Post 3	141	141	100	(97.4; 100)	142.2	(116.4; 173.7)
R2	Screening	124	87	70.2	(61.3; 78.0)	0.8	(0.7; 0.9)
	Post 3	123	123	100	(97.0; 100)	188.5	(156.5; 227.0)
R3	Screening	137	80	58.4	(49.7; 66.7)	0.6	(0.6; 0.8)
	Post 3	136	135	99.3	(96.0; 100)	205.5	(167.3; 252.5)
C1	Screening	136	84	61.8	(53.0; 70.0)	0.6	(0.6; 0.7)
	Post 3	135	16	11.9	(6.9; 18.5)	0.3	(0.3; 0.3)
C2	Screening	118	75	63.6	(54.2; 72.2)	0.7	(0.6; 0.8)
	Post 3	118	12	10.2	(5.4; 17.1)	0.3	(0.3; 0.4)

Group R1 received RTS,S/AS01 + (DTaP/Hib + tOPV + PHiD-CV), and HRV 2 weeks later.

Group R2 received RTS,S/AS01 + (DTaP/Hib + tOPV + HRV), and PHiD-CV 2 weeks later.

Group R3 received RTS,S/AS01 + (DTaP/Hib + tOPV), and (PHiD-CV + HRV) 2 weeks later.

Group C1 received HBV + (DTaP/Hib + tOPV + PHiD-CV), and HRV 2 weeks later.

Group C2 received HBV + (DTaP/Hib + tOPV + HRV), and PHiD-CV 2 weeks later.

N = number of participants with available results, n/% = number/percentage of participants with concentration ≥ specified value, CS = circumsporozoite protein, 95% CI = 95% confidence interval, Screening = Pre-vaccination, Post 3 = one month post-dose 3, DTaP/Hib = diphtheria-tetanus-acellular pertussis-Haemophilus influenzae type-b-conjugate vaccine, tOPV = trivalent oral poliovirus vaccine, PHiD-CV = 10-valent pneumococcal non-typeable *Haemophilus influenzae* protein D conjugate vaccine, HRV = human rotavirus vaccine.

* The adapted cohort is the according-to-protocol cohort at each individual time point.

### Immune response to co-administered vaccines

#### Pneumococcal conjugate vaccine

The pre-specified non-inferiority criterion was reached for 9/10 vaccine pneumococcal serotypes (Table 2). For serotype 18C, the upper limit of the 95% CI around the antibody GMC ratio (group C1 over group R1) was 2.38 which was above the pre-defined limit for non-inferiority of 2.

**Figure 2.**
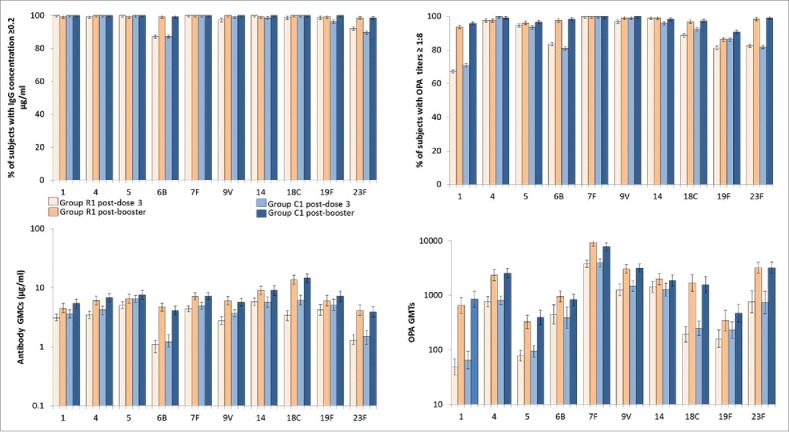
Anti-pneumococcal antibody concentrations and OPA titers after primary vaccination with PHiD-CV (with or without RTS,S/AS01 co-administration) and booster vaccination (according to protocol immunogenicity cohort at each time point). Vertical lines indicate 95% confidence intervals, OPA = opsonophagocytic activity, PHiD-CV = 10-valent pneumococcal non-typeable *Haemophilus influenzae* protein D conjugate vaccine, GMC/T = geometric mean antibody concentration/titer.

One month post-dose 3, for each of the vaccine serotypes 97.2% to 100% of children in the group R1 and in the group C1 had antibody concentrations ≥0.2 μg/ml, except for serotypes 6B (87.2% and 87.4% in the respective groups) and 23F (92.1% and 89.6%, respectively) (Fig. 2, Supplement Table 3). For each vaccine serotype except 18C, the antibody GMCs were in the same ranges in the groups R1 and C1 (Fig. 2).

One month post-dose 3, for each vaccine serotype, between 81.0% and 100% of children in both groups had opsonophagocytic activity (OPA) titers ≥8, except for serotype 1 (67.4% in the group R1 and 71.0% in the group C1) (Fig. 2, Supplement Table 4). No consistent trend was observed between groups in terms of post-primary OPA GMTs.

After the PHiD-CV booster dose, for each vaccine serotype 98.4% to 100% of participants in the groups R1 and C1 had antibody concentrations ≥0.2 μg/ml (Fig. 2). For each vaccine serotype 96.1% to 100% of children had OPA titers ≥8, except for serotypes 1 (93.8% and 95.9% in the respective groups) and 19F (86.2% and 90.9%, respectively). Post-booster antibody GMCs and OPA GMTs were higher than after primary vaccination for all vaccine serotypes. No consistent trend was observed in terms of impact of RTS,S/AS01 co-administration on antibody GMCs and OPA GMTs after the PHiD-CV booster dose.

One month post-dose 3 all infants had anti-protein D antibody concentrations ≥100 EU/mL. The anti-Protein D antibody GMCs were similar in the two groups (Supplement Table 5).

**Table 5.** Anti-rotavirus antibody seropositivity rates and geometric mean antibody concentrations one month post-dose 2 (according to protocol immunogenicity cohort).

		≥ 20 U/ml			GMC	
Group	N	n	%	(95% CI)	value	(95% CI)
R2	120	44	36.7	(28.1; 45.9)	24.9	(19.3; 32.0)
C2	116	43	37.1	(28.3; 46.5)	27.6	(20.8; 36.5)

Group R2 received RTS,S/AS01 + (DTaP/Hib + tOPV + HRV), and PHiD-CV 2-weeks later. Group C2 received HBV + (DTaP/Hib + tOPV + HRV), and PHiD-CV 2 weeks later.

GMC = geometric mean antibody concentrations calculated on all participants.

N = number of participants with available results.

n/% = number/percentage of participants with concentrations equal to or above specified value.

95% CI = 95% confidence interval.

DTaP/Hib = diphtheria-tetanus-acellular pertussis-Haemophilus influenzae type-b-conjugate vaccine.

tOPV = trivalent oral poliovirus vaccine.

PHiD-CV = 10-valent pneumococcal non-typeable *Haemophilus influenzae* protein D conjugate vaccine, HRV = human rotavirus vaccine.

#### Human rotavirus vaccine

Non-inferiority for anti-HRV responses between the two groups that received HRV with and without RTS,S/AS01 (R2 and C2 respectively) was demonstrated in terms of the pre-specified criteria (Table 2). The percentage of children in each group who were seropositive for anti-rotavirus IgA antibodies after two doses of HRV was 36.7% in the group R2 and 37.1% in the group C2 (Table 5).

#### Acellular pertussis antigens

Non-inferiority for pertussis antibody responses in the groups R and C was demonstrated in terms of the pre-specified criteria (Table 2). All participants in both groups R and C were seropositive for antibodies to the three pertussis vaccine antigens (pertussis toxin, filamentous haemagglutinin and pertactin) one month after dose 3. In addition, 98.0% to 100% of participants had a vaccine response (defined as seroconversion in initially seronegative infants or an antibody concentrations post-vaccination at least equal to the concentrations prior to vaccination in initially seropositive infants) (Supplement Table 6).

**Table 6.** Solicited local (by administered product and at any injection site) and systemic symptoms over 7 days post-primary vaccination (Days 0–6) overall doses (Total vaccinated cohort^*^).

			Group R1				Group R2				Group R3				Group C1				Group C2			
Symptom	Vaccine	Type	N	n	%	(95% CI)	N	n	%	(95% CI)	N	n	%	(95% CI)	N	n	%	(95% CI)	N	n	%	(95% CI)
Pain	HBV	All	—	—	—	—	—	—	—	—	—	—	—	—	420	44	10.5	(7.7; 13.8)	382	23	6.0	(3.9; 8.9)
		Grade 3	—	—	—	—	—	—	—	—	—	—	—	—	420	0	0.0	(0.0; 0.9)	382	0	0.0	(0.0; 1.0)
	DTaP/Hib	All	424	64	15.1	(11.8; 18.9)	394	42	10.7	(7.8; 14.1)	423	44	10.4	(7.7; 13.7)	423	47	11.1	(8.3; 14.5)	385	27	7.0	(4.7; 10.0)
		Grade 3	424	1	0.2	(0.0; 1.3)	394	0	0.0	(0.0; 0.9)	423	0	0.0	(0.0; 0.9)	423	0	0.0	(0.0; 0.9)	385	0	0.0	(0.0; 1.0)
	RTS,S/AS01	All	424	56	13.2	(10.1; 16.8)	394	37	9.4	(6.7; 12.7)	423	49	11.6	(8.7; 15.0)	—	—	—	—	—	—	—	—
		Grade 3	424	0	0.0	(0.0; 0.9)	394	0	0.0	(0.0; 0.9)	423	1	0.2	(0.0; 1.3)	—	—	—	—	—	—	—	—
	PHiD-CV	All	424	64	15.1	(11.8; 18.9)	—	—	—	—	—	—	—	—	423	48	11.3	(8.5; 14.8)	—	—	—	—
		Grade 3	424	0	0.0	(0.0; 0.9)	—	—	—	—	—	—	—	—	423	0	0.0	(0.0; 0.9)	—	—	—	—
Redness (mm)	HBV	All	—	—	—	—	—	—	—	—	—	—	—	—	420	4	1.0	(0.3; 2.4)	382	1	0.3	(0.0; 1.4)
		>20.0	—	—	—	—	—	—	—	—	—	—	—	—	420	0	0.0	(0.0; 0.9)	382	0	0.0	(0.0; 1.0)
	DTaP/Hib	All	424	4	0.9	(0.3; 2.4)	394	1	0.3	(0.0; 1.4)	423	4	0.9	(0.3; 2.4)	423	5	1.2	(0.4; 2.7)	385	1	0.3	(0.0; 1.4)
		>20.0	424	0	0.0	(0.0; 0.9)	394	0	0.0	(0.0; 0.9)	423	0	0.0	(0.0; 0.9)	423	0	0.0	(0.0; 0.9)	385	0	0.0	(0.0; 1.0)
	RTS,S/AS01	All	424	3	0.7	(0.1; 2.1)	394	0	0.0	(0.0; 0.9)	423	3	0.7	(0.1; 2.1)	—	—	—	—	—	—	—	—
		>20.0	424	0	0.0	(0.0; 0.9)	394	0	0.0	(0.0; 0.9)	423	0	0.0	(0.0; 0.9)	—	—	—	—	—	—	—	—
	PHiD-CV	All	424	7	1.7	(0.7; 3.4)	—	—	—	—	—	—	—	—	423	6	1.4	(0.5; 3.1)	—	—	—	—
		>20.0	424	0	0.0	(0.0; 0.9)	—	—	—	—	—	—	—	—	423	0	0.0	(0.0; 0.9)	—	—	—	—
Swelling (mm)	HBV	All	—	—	—	—	—	—	—	—	—	—	—	—	420	15	3.6	(2.0; 5.8)	382	3	0.8	(0.2; 2.3)
		>20.0	—	—	—	—	—	—	—	—	—	—	—	—	420	0	0.0	(0.0; 0.9)	382	0	0.0	(0.0; 1.0)
	DTaP/Hib	All	424	13	3.1	(1.6; 5.2)	394	5	1.3	(0.4; 2.9)	423	10	2.4	(1.1; 4.3)	423	19	4.5	(2.7; 6.9)	385	10	2.6	(1.3; 4.7)
		>20.0	424	0	0.0	(0.0; 0.9)	394	0	0.0	(0.0; 0.9)	423	0	0.0	(0.0; 0.9)	423	0	0.0	(0.0; 0.9)	385	0	0.0	(0.0; 1.0)
	RTS,S/AS01	All	424	9	2.1	(1.0; 4.0)	394	4	1.0	(0.3; 2.6)	423	9	2.1	(1.0; 4.0)	—	—	—	—	—	—	—	—
		>20.0	424	0	0.0	(0.0; 0.9)	394	0	0.0	(0.0; 0.9)	423	0	0.0	(0.0; 0.9)	—	—	—	—	—	—	—	—
	PHiD-CV	All	424	9	2.1	(1.0; 4.0)	—	—	—	—	—	—	—	—	423	16	3.8	(2.2; 6.1)	—	—	—	—
		>20.0	424	0	0.0	(0.0; 0.9)	—	—	—	—	—	—	—	—	423	0	0.0	(0.0; 0.9)	—	—	—	—
Pain	Any injection site	All	424	85	20.0	(16.3; 24.2)	394	52	13.2	(10.0; 16.9)	423	66	15.6	(12.3; 19.4)	423	71	16.8	(13.3; 20.7)	385	31	8.1	(5.5; 11.2)
		Grade 3	424	1	0.2	(0.0; 1.3)	394	0	0.0	(0.0; 0.9)	423	1	0.2	(0.0; 1.3)	423	0	0.0	(0.0; 0.9)	385	0	0.0	(0.0; 1.0)
Redness (mm)		All	424	9	2.1	(1.0; 4.0)	394	1	0.3	(0.0; 1.4)	423	5	1.2	(0.4; 2.7)	423	11	2.6	(1.3; 4.6)	385	1	0.3	(0.0; 1.4)
		>20.0	424	0	0.0	(0.0; 0.9)	394	0	0.0	(0.0; 0.9)	423	0	0.0	(0.0; 0.9)	423	0	0.0	(0.0; 0.9)	385	0	0.0	(0.0; 1.0)
Swelling (mm)		All	424	20	4.7	(2.9; 7.2)	394	7	1.8	(0.7; 3.6)	423	16	3.8	(2.2; 6.1)	423	30	7.1	(4.8; 10.0)	385	11	2.9	(1.4; 5.1)
		>20.0	424	0	0.0	(0.0; 0.9)	394	0	0.0	(0.0; 0.9)	423	0	0.0	(0.0; 0.9)	423	0	0.0	(0.0; 0.9)	385	0	0.0	(0.0; 1.0)
Drowsiness		All	424	10	2.4	(1.1; 4.3)	394	2	0.5	(0.1; 1.8)	423	6	1.4	(0.5; 3.1)	423	7	1.7	(0.7; 3.4)	385	0	0.0	(0.0; 1.0)
		Grade 3	424	0	0.0	(0.0; 0.9)	394	0	0.0	(0.0; 0.9)	423	0	0.0	(0.0; 0.9)	423	0	0.0	(0.0; 0.9)	385	0	0.0	(0.0; 1.0)
Irritability/fussiness		All	424	33	7.8	(5.4; 10.8)	394	21	5.3	(3.3; 8.0)	423	33	7.8	(5.4; 10.8)	423	25	5.9	(3.9; 8.6)	385	6	1.6	(0.6; 3.4)
		Grade 3	424	0	0.0	(0.0; 0.9)	394	0	0.0	(0.0; 0.9)	423	0	0.0	(0.0; 0.9)	423	0	0.0	(0.0; 0.9)	385	0	0.0	(0.0; 1.0)
Loss of appetite		All	424	9	2.1	(1.0; 4.0)	394	2	0.5	(0.1; 1.8)	423	4	0.9	(0.3; 2.4)	423	8	1.9	(0.8; 3.7)	385	1	0.3	(0.0; 1.4)
		Grade 3	424	0	0.0	(0.0; 0.9)	394	0	0.0	(0.0; 0.9)	423	0	0.0	(0.0; 0.9)	423	0	0.0	(0.0; 0.9)	385	0	0.0	(0.0; 1.0)
Fever (axillary route)		≥37.5°C	424	112	26.4	(22.3; 30.9)	394	54	13.7	(10.5; 17.5)	423	60	14.2	(11.0; 17.9)	423	59	13.9	(10.8; 17.6)	385	30	7.8	(5.3; 10.9)
		>39.0°C	424	5	1.2	(0.4; 2.7)	394	0	0.0	(0.0; 0.9)	423	3	0.7	(0.1; 2.1)	423	1	0.2	(0.0; 1.3)	385	4	1.0	(0.3; 2.6)

Group R1 = RTS,S/AS01 + (DTaP/Hib + tOPV + PHiD-CV), and HRV 2 weeks later.

Group R2 = RTS,S/AS01 + (DTaP/Hib + tOPV + HRV), and PHiD-CV 2 weeks later.

Group R3 = RTS,S/AS01 + (DTaP/Hib + tOPV), and PHiD-CV + HRV 2 weeks later.

Group C1 = HBV + (DTaP/Hib + tOPV + PHiD-CV), and HRV 2 weeks later.

Group C2 = HBV+ (DTaP/Hib + tOPV + HRV), and PHiD-CV 2 weeks later.

N = number of administered doses, n/% = number/percentage of doses followed by at least one type of symptom, 95% CI = Exact 95% confidence interval.

Grade 3: Pain = Cries when limb is moved/spontaneously painful. Irritability/Fussiness = Crying that cannot be comforted/prevents normal activity, Drowsiness = Drowsiness that prevents normal activity, Loss of appetite = Not eating at all.

DTaP/Hib = diphtheria-tetanus-acellular pertussis-Haemophilus influenzae type-b-conjugate vaccine.

tOPV = trivalent oral poliovirus vaccine.

PHiD-CV = 10-valent pneumococcal non-typeable *Haemophilus influenzae* protein D conjugate vaccine.

HRV = human rotavirus vaccine.

* The analysis of safety was done considering the treatment actually administered. Three infants in the group C1 inadvertently received co-administered RTS,S/AS01 and three in the group C2 inadvertently received co-administered RTS,S/AS01 + PHiD-CV. Pain at the RTS,S/AS01 injection site was reported for one subject in the group R1. No other symptoms were reported for these subjects at the RTS,S/AS01 or PHiD-CV injection sites.

### Safety

Solicited local and systemic reactogenicity

Pain was the most frequently reported local symptom at each injection site in all treatment groups during the 7-day follow-up after each primary vaccination with RTS,S/AS01 or HBV (and co-administered vaccines) (Table 6). Pain tended to be more frequent in the groups that received either RTS,S/AS01 or PHiD-CV, and to have the highest frequency in the group that received both vaccines together (Table 6). Grade 3 local symptoms were rare (0%-0.2% of doses). The occurrence of pain, redness and swelling was similar at the DTaP/Hib, PHiD-CV and RTS,S/AS01 injection sites (Table 6). Local reactogenicity did not increase with consecutive doses (Supplement Table 7).

Fever was the most frequently reported general symptom in all treatment groups during the 7-day follow-up period after each RTS,S/AS01 or HBV primary vaccination. Fever was reported after a minimum of 7.8% (group C2) and a maximum of 26.4% (group R1) of all doses. Fever tended to be more frequent in the groups that received either RTS,S/AS01 or PHiD-CV, and had the highest frequency in the group that received both vaccines together (Supplement Fig. 2). The occurrence and severity of fever did not increase markedly with consecutive doses in any group (Supplement Table 8). Fever with temperature >39.0°C was reported infrequently (≤1.2% of doses).

Other systemic adverse events (drowsiness, irritability/fussiness, and loss of appetite) were reported after a maximum of 7.8% of doses in any co-administration group, and none of them was grade 3 (Table 6).

### Unsolicited adverse events

Other (unsolicited) adverse events that occurred until 30 days (Day 0–29) after each primary dose of RTS,S/AS01 or HBV vaccine were reported for 75.5%-85.2% of children in each treatment group. The most frequently reported adverse events were malaria, gastroenteritis, bronchitis, pharyngitis, rhinitis, upper respiratory tract infection and erythema. Adverse events considered by the investigator to be causally related to vaccination were reported for 0.7% to 4.3% of children. All related adverse events were pyrexia and/or pain. Grade 3 unsolicited adverse events were reported for eight children (no more than two in any treatment group). None were considered to be causally related to vaccination. No seizures or cases of meningitis were reported during the 30 days follow-up period after any RTS,S/AS01 or HBV dose.

#### SAEs until 6 months post-dose 3

SAEs occurring until 6 months post-dose 3 were reported for 23 children of which five were fatal. Bronchopneumonia and gastroenteritis were reported more than once in any group. None of the SAEs were considered by the investigator to be vaccine-related.

#### Fatal and related SAEs

During the entire study period until month 26 eight deaths were reported (Supplement Table 1). None of the SAEs were considered by the investigator to be related to vaccination. No potential immune-mediated diseases (pIMDs) were reported during the entire study period.

## Discussion

The primary study objective showed that three priming doses of RTS,S/AS01 induced an immune response against HBsAg that was non-inferior to three priming doses of a licensed HBV vaccine. The anti-HBs antibody GMC after three doses of RTS,S/AS01 was approximately 17-fold higher than after three doses of a licensed HBV vaccine. Current recommendations indicate that individuals who achieve effective primary vaccination against HBV develop immune memory and do not need further booster doses.^7^^,^^8^ It is therefore not clear whether the higher antibody GMCs following RTS,S/AS01 compared to a licensed HBV vaccine will also result in clinical benefits in terms of less non-responders or longer-term protection. This finding will be investigated further with assessment of anti-HBs antibody persistence and immune memory to HBV at 4–5 years of age through administration of an HBV booster dose, and will be reported separately.

RTS,S/AS01 induced anti-CS antibodies in all vaccine recipients except one. This study indicated a trend towards a lower anti-CS antibody GMC when RTS,S/AS01 was co-administered with PHiD-CV or HRV (groups R1 and R2) than when administered alone (group R3). The clinical relevance of this observation is currently unknown, but anti-CS antibody GMCs in the present study are within the range reported in previous studies of RTS,S/AS01 in infants in Africa, in whom vaccine efficacy was demonstrated.^9^ While anti-CS antibodies were shown to be associated with protection against clinical malaria, today no threshold level of protection has been identified.^10^

This study builds on previous co-administration studies of RTS,S/AS01 and DTwP-combination vaccines by investigating co-administration with an aP-containing vaccine, as well as PCV and rotavirus vaccines recommended for administration during infancy. Post-primary antibody GMCs were slightly lower for most pneumococcal vaccine serotypes when PHiD-CV was co-administered with RTS,S/AS01 as compared to co-administration with HBV, while being within the range of pneumococcal antibody GMCs reported in previous studies of PHiD-CV conducted in Africa.^11^^,^ ^12^ Consistent with previous experience with PCV, antibody responses to PHiD-CV vaccination were lowest for serotypes 6B and 23F in both groups,^13^ but the pre-defined non-inferiority criterion based on the post-primary vaccination antibody GMC ratios was met for all pneumococcal vaccine serotypes except serotype 18C. However, since the percentage of participants with antibody concentrations against 18C ≥0.2 μg/ml was very high (98.6% in the group R1 and 100% in the group C1) and a functional OPA response was measured for all vaccine serotypes in both groups, including 18C, this difference is unlikely to have clinical importance. Moreover, for all vaccine serotypes the booster dose of PHiD-CV induced antibody concentrations ≥0.2 μg/ml in 98% to 100% of participants, with marked increases in antibody GMCs and OPA GMTs for all serotypes, indicative of effective immune priming in both groups.

A booster dose of PCV is not currently included in many immunization schedules in developing countries. However, in 2012 the WHO recognized that booster vaccination may be beneficial in settings where children are at particular risk of invasive pneumococcal disease.^14^ The arguments for a booster dose are particularly compelling in Africa where serotype 1 is one of the most frequent causes of invasive pneumococcal disease, and where immunity to serotype 1 wanes rapidly in the absence of a booster dose.^15^^,^ ^16^ In the present study and consistent with previous reports, the percentage of participants with OPA titers ≥8 after primary vaccination was lower for serotype 1 than for other serotypes in both groups, even though 99% to 100% of all subjects had antibody concentrations ≥0.2 μg/ml for serotype 1.^13^ The PHiD-CV booster dose markedly increased serotype 1 OPA GMTs in both groups. Our study therefore supports the likely benefit of a PCV booster dose in the African context. Booster vaccination is currently administered in South Africa, using the alternative 2-dose primary and early (9 months of age) booster schedule also advocated by WHO.^14^^,^ ^17^

Pre-defined criteria for non-inferiority for immune responses to co-administered HRV were met. Seroconversion rates to HRV were low in both groups, and low responses to HRV in African children have been reported previously.^18^ However, rotavirus vaccine effectiveness in African countries has been demonstrated and appears to be sustained.^19^

RTS,S/AS01 was well tolerated. No SAEs or fatalities were considered to be vaccine-related. The occurrence of fever was more frequent in groups receiving either RTS,S/AS01 or PHiD-CV, and was the highest in the group receiving both vaccines together, for which fever was similar to a previous study in infants in which RTS,S/AS01 was co-administered with DTPw-HBV/Hib vaccine.^20^ Fever >39.0°C was uncommon and no seizures were reported within 30 days of RTS,S/AS01 immunization.

At study initiation we were unable to access a DTwP combination that did not include HBsAg, and were therefore compelled to use DTaP in order to evaluate the primary study endpoint. DTwP is used more frequently than DTaP in Africa and DTwP has been successfully co-administered with RTS,S/AS01 in large numbers of infants.^3^^,^ ^21^ Hepatitis B vaccines are highly immunogenic regardless of whether they are administered or combined with DTaP or DTwP, thus the findings of our study are broadly applicable, especially with regard to the primary endpoint. In view of the higher reactogenicity of DTwP compared with DTaP vaccines, the occurrence of fever when RTS,S/AS01 is co-administered with PCVs and DTwP would need further investigation should RTS,S/AS01 be implement in infants eligible for DTwP and PCV vaccination.

In a large Phase III study,^2^ meningitis (of any etiology) was reported as a SAE up to 48 months after dose 1 more frequently in 5–17 months old vaccinated with RTS,S/AS01 (21 cases per 5,948 children) compared to the rabies vaccine control group (1 case per 2,974 children). A causal relationship to the vaccine has not been established. No cases of meningitis were recorded during the present study, but it should be considered that the study sample size was too small to detect the occurrence of uncommon adverse events.

While we made every effort to conduct a study that reflected standard immunization practices in Africa, the study has potential limitations. Because the DTaP/Hib vaccine was not indicated for use below 8 weeks of age, we employed an 8, 12 and 16 weeks schedule rather than the standard 6, 10 and 14 weeks schedule used in Africa to conform with vaccine usage recommendations. In programmatic implementation there is an age range at which each scheduled dose may be administered. Therefore the study schedule can be considered broadly applicable.

In conclusion, RTS,S/AS01 induced an immune response to HBV that was at least as good as that induced by a licensed HBV vaccine. RTS,S/AS01 should however only be used for the prevention of HBV in settings where prevention against malaria is sought.^22^ Immune responses to RTS,S/AS01 co-administered with DTaP, tOPV, PHiD-CV and HRV were satisfactory, and RTS,S/AS01 co-administered with these vaccines had an acceptable safety profile.

**Figure 3.**
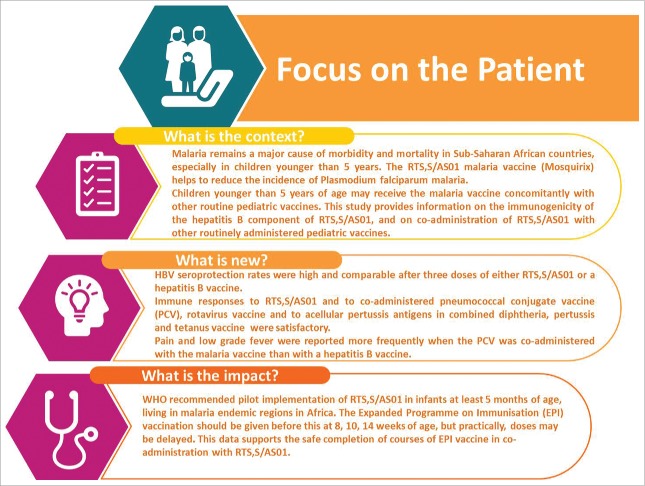
Focus on Patient Section.

Fig. 3 represents a Focus on Patient Section, which elaborates on the research clinical relevance to be shared to patients by Health Care Professionals.

## Methods

### Study design and participants

The study was open, randomized (113681, www.clinicaltrials.gov NCT01345240) and conducted at the Institut de Recherche en Sciences de la Santé/Centre Medical Sainte Camille de Nanoro in Burkina Faso, and the Malaria Research Centre Agogo Presbyterian Hospital/Kwame Nkrumah University of Science and Technology in Ghana. Participants were healthy infants 8–12 weeks of age born to mothers who were HBsAg seronegative and negative for antibodies to Human Immunodeficiency Virus. Written informed consent was obtained from the children's parents or guardians. The study was undertaken in accordance with Good Clinical Practice guidelines and Declaration of Helsinki. An Independent Data Monitoring Committee monitored the study and reviewed study endpoints and safety data. See Supplement 9 for details on study oversight, consent procedures, and screening activities.

### Randomization and Vaccines

Infants were randomized in a 1:1:1:1:1 ratio to five treatment groups (Table 1). Randomization was performed at GSK using SAS® (Cary, NC, USA). Treatment allocation at the study site was done centrally on the internet. The randomization algorithm used a minimization procedure accounting for center.

All participants received measles and yellow fever vaccines at 9 months of age and booster doses of PHiD-CV and DTaP/Hib at 18 months of age. Vaccine composition is provided in Supplement 9. All vaccines were manufactured by GSK.

Persistence of anti-HBs and anti-CS antibodies was to be assessed annually up to 48 months after primary vaccination. Persistence of anti-HBs and anti-CS antibodies and the immunogenicity of a booster dose of HBV vaccine administered 4 years after primary vaccination will be reported at study completion.

### Objectives

The primary study objective was to demonstrate the non-inferiority of RTS,S/AS01 to HBV vaccine in terms of anti-HBs seroprotection rates one month post-dose 3 (group R compared to group C). Secondary objectives reported here are demonstration of the non-inferiority of the immune response to PHiD-CV, HRV and aP antigens when co-administered with RTS,S/AS01 to co-administration with a licensed HBV vaccine; anti-CS antibodies when RTS,S/AS01 is co-administered with PHiD-CV or HRV; assessment of a booster dose of DTaP/Hib and PHiD-CV administered during the second year of life; and immunogenicity and safety of the study vaccines.

### Immunogenicity assessment

Anti-HBs antibodies were measured using a Chemiluminescence Immunoassay (CLIA) (*Centaur*™, Siemens) with a cut-off of 6.2 mIU/ml. An anti-HBs concentrations equal to or above 10mIU/ml is considered seroprotective.^8^ Serological assays for the determination of antibodies against CS, HBs RF1, protein D, pertussis antigens and rotavirus were performed by ELISA at a GSK or designated laboratory using standard methods. The assay cut-off for antibodies was 0.5 EU/ml for CS, 5 EU/ml for each pertussis antigen, 33 EU/ml for HBs RF1, 100 EU/ml for Protein D and 20 U/ml for HRV IgA.

Serum anti-pneumococcal IgG concentrations were measured by 22F-inhibition ELISA with an assay cut-off of 0.05 µg/ml for each pneumococcal serotype. It has been established that an antibody threshold value of 0.2 µg/mL using the 22F- ELISA is equivalent to the WHO recommended reference value of 0.35 µg/mL using the non-22F ELISA^23^

OPA of anti-pneumococcal antibodies was measured by a killing assay using a HL60 cell line.^24^ The results are presented as the dilution of serum (opsonic titer) able to induce 50% killing of live pneumococci under the assay conditions. The assay cut-off was at an opsonic titer of 8.

Refer to Supplement 9 for a description of the blood sampling schedule.

### Assessment of reactogenicity and safety

Participants were visited daily for 7 days following the administration of each dose of RTS,S/AS01 or HBV and information on the occurrence of specific local and general symptoms was recorded by study staff. All adverse events starting within 30 days following administration of each dose of RTS,S/AS01 or HBV were recorded at the next vaccination visit. All SAEs were recorded from dose 1 until 6 months after the last dose of RTS,S/AS01 or HBV vaccine at any time during the study, or at the next vaccination visit. At each study contact, parents were encouraged by study staff to present the child to a healthcare facility at any time they felt their child was unwell. SAEs that were related to study participation and fatal SAEs were captured during the whole study period. See supplement 9 for a description of causality and severity assessments.

Adverse events of special interest were seizures, rashes and mucocutaneous lesions occurring within a 30-day period of vaccination, and pIMDs occurring over the entire study period. Seizures and pIMDs were to be reported as SAEs.

### Statistical analyses

The primary cohort for the analysis of immunogenicity was the according-to-protocol immunogenicity cohort, which comprised all eligible participants who complied with study procedures and for whom data concerning immunogenicity endpoint measures are available. Non-inferiority of the anti-HBs responses was concluded if the upper limit of the 95% CI around the difference in the anti-HBs seroprotection rate in the group C minus the rate in the group R one month post-dose 3 was <5%. Criteria for non-inferiority of the responses to each PHiD-CV vaccine serotype, HRV and aP antigens one month post-dose 3 are summarized in Table 2.

The analysis of safety was performed on the total vaccinated cohort, which included all participants who had received at least one dose of study vaccine.

The analysis of the primary and secondary inferential endpoints defined in Table 1 was performed on data collected up to 1 month post-dose 3 of RTS,S/AS01 or HBV. The primary safety analysis was conducted on data collected until 6 months after the last dose of RTS,S/AS01 or HBV. The immunogenicity follow-up analysis conducted at month 26 assessed the response to PHiD-CV before and one month after the PHiD-CV + DTaP/Hib booster dose, anti-CS antibodies until 1 year post primary vaccination (month 14), and anti-HBs antibodies and safety data until 2 years post-primary vaccination (month 26).

The study was powered to have 92% power to demonstrate non-inferiority of the anti-HBs response (primary endpoint), and at least 80% power to conclude on secondary non-inferiority endpoints for PCV and HRV.

## Supplementary Material

KHVI_A_1442996_Supplemental.docx

## Trademark statement

ENGERIX, MOSQUIRIX, ROTARIX, SYNFLORIX, INFANRIX AND POLIO SABIN are trademarks owned by, or licensed to, the GSK group of companies.
