# Supplementary material for: Immune response to the hepatitis B antigen in the RTS,S/AS01 malaria vaccine, and co-administration with pneumococcal conjugate and rotavirus vaccines in African children: A randomized controlled trial
Source: Hum Vaccin Immunother. 2018 Apr 13;14(6):1489–500. doi: 10.1080/21645515.2018.1442996 (PMC6037440; doi:10.1080/21645515.2018.1442996)
Supplement: KHVI_A_1442996_Supplemental.docx [file khvi-14-06-1442996-s001.docx]

**Supplementary material**

**Supplement Table 1. Fatal Serious Adverse Events reported from study commencement until Month 26 (Total vaccinated cohort)**

| **Group** | **Age at onset (Month)** | **Sex** | **Preferred term** | **System Organ Class** | **Vaccination *** | **Day of  onset** | **Duration**  **(days)** |
| --- | --- | --- | --- | --- | --- | --- | --- |
| R1 | 2 | Male | Failure to thrive | Metabolism and nutrition disorders | 1 | 10 | 7 |
|  |  |  | Gastroenteritis | Infections and infestations | 1 | 10 | 7 |
|  |  |  | Malnutrition | Metabolism and nutrition disorders | 1 | 10 | 7 |
| R2 | 8 | Male | Pyrexia | General disorders and administration site conditions | 6 | 137 | 1 |
|  | 9 | Male | Accident | Injury, poisoning and procedural complications | 6 | 141 | 1 |
| R3 | 3 | Male | Bronchitis | Infections and infestations | 5 | 2 | 2 |
|  | 6 | Male | Bronchopneumonia | Infections and infestations | 6 | 76 | 16 |
|  | 7 |  | Hypertrophic cardiomyopathy | Congenital, familial and genetic disorders | 6 | 89 | 3 |
| C1 | 10 | Female | Anaemia | Blood and lymphatic system disorders | 5 | 184 | 1 |
|  |  |  | Bacterial sepsis | Infections and infestations | 5 | 184 | 1 |
|  |  |  | Malaria | Infections and infestations | 5 | 184 | 1 |
|  | 10 | Female | Gastrointestinal haemorrhage | Gastrointestinal disorders | 5 | 192 | 1 |
|  |  |  | Sepsis | Infections and infestations | 5 | 192 | 1 |
| C2 | 18 | Female | Anaemia | Blood and lymphatic system disorders | 7 | 29 | 1 |
|  |  |  | Bronchiolitis | Infections and infestations | 7 | 27 | 3 |

Group R1 received RTS,S/AS01 + (DTPa/Hib + tOPV + PHiD-CV), and HRV 2 weeks later

Group R2 received RTS,S/AS01 + (DTPa/Hib + tOPV + HRV), and PHiD-CV 2 weeks later

Group R3 received RTS,S/AS01 + (DTPa/Hib + tOPV), and (PHiD-CV + HRV) 2 weeks later

Group C1 received HBV + (DTPa/Hib + tOPV + PHiD-CV), and HRV 2 weeks later

Group C2 received HBV + (DTPa/Hib + tOPV + HRV), and PHiD-CV 2 weeks later

DTaP/Hib = diphtheria-tetanus-acellular-pertussis-Haemophilus influenzae type-b-conjugate vaccine, tOPV = trivalent oral poliovirus vaccine, PHiD-CV = 10-valent pneumococcal non-typeable Haemophilus influenzae protein D conjugate vaccine, HRV = human rotavirus vaccine, HBV = hepatitis B vaccine

* See Supplementary Figure 1 for a visual explanation of vaccinations administered to each group.

**Supplement Table 2. Demographic characteristics at enrolment (Total vaccinated cohort)**

| **Characteristics** | **Categories** | **R1 N = 142** | **R2 N = 142** | **R3  N = 141** | **C1 N = 141** | **C2 N = 139** |
| --- | --- | --- | --- | --- | --- | --- |
| Age at first vaccination (weeks) | Mean (SD) | 8.4 (0.8) | 8.3 (0.6) | 8.3 (0.7) | 8.3 (0.7) | 8.3 (0.)7 |
|  | Range | 8.0-12.0 | 8.0-11.0 | 8.0-11.0 | 8.0-12.0 | 8.0-11.0 |
| Gender | Female | 59 (41.5) | 69 (48.6) | 67 (47.5) | 81 (57.4) | 63 (45.3) |
|  | Male | 83 (58.5) | 73 (51.4) | 74 (52.5) | 60 (42.6) | 76 (54.7) |
| Height for age z-score | Mean (SD) | -0.8 (1.3) | -0.8 (1.2) | -0.8 (1.3) | -0.8 (1.2) | -0.7 (1.2) |
|  | Range | -4.2-3.6 | -3.9-2.6 | -3.2-5.4 | -4.2-1.8 | -3.5-3.7 |
| Weight for age z-score | Mean (SD) | -0.7 (1.0) | -0.4 (0.9) | -0.5 (0.9) | -0.5 (1.0) | -0.4 (1.0) |
|  | Range | -3.9-1.9 | -3.4-1.9 | -3.5-1.1 | -3.1-2.2 | -3.0-2.4 |

Group R1 received RTS,S/AS01 + (DTPa/Hib + tOPV + PHiD-CV), and HRV 2 weeks later

Group R2 received RTS,S/AS01 + (DTPa/Hib + tOPV + HRV), and PHiD-CV 2 weeks later

Group R3 received RTS,S/AS01 + (DTPa/Hib + tOPV), and (PHiD-CV + HRV) 2 weeks later

Group C1 received HBV + (DTPa/Hib + tOPV + PHiD-CV), and HRV 2 weeks later

Group C2 received HBV + (DTPa/Hib + tOPV + HRV), and PHiD-CV 2 weeks later

N = number of subjects

SD = standard deviation

DTaP/Hib = diphtheria-tetanus-acellular-pertussis-Haemophilus influenzae type-b-conjugate vaccine, tOPV = trivalent oral poliovirus vaccine, PHiD-CV = 10-valent pneumococcal non-typeable Haemophilus influenzae protein D conjugate vaccine, HRV = human rotavirus vaccine, HBV = hepatitis B vaccine.

**Supplement Table 3.** **Percentages of infants with serotype-specific pneumococcal antibody titers ≥0.2 µg/ml and geometric mean antibody concentrations one month post-dose 3 and one month post-booster at 18 months of age (Adapted ATP immunogenicity cohort).**

|  | | | | **≥ 0.2 µg/ml** | | | | **GMC** | | |
| --- | --- | --- | --- | --- | --- | --- | --- | --- | --- | --- |
|  | | | |  | | **95% CI** | |  | **95% CI** | |
| **Antibody** | **Group** | **Timing** | **N** | **n** | **%** | **LL** | **UL** | **value** | **LL** | **UL** |
| anti-1 | R1 | Post 3 | 141 | 141 | 100 | 97.4 | 100 | 3.1 | 2.8 | 3.6 |
|  |  | Post B | 133 | 132 | 99.2 | 95.9 | 100 | 4.5 | 3.8 | 5.4 |
|  | C1 | Post 3 | 135 | 135 | 100 | 97.3 | 100 | 3.6 | 3.1 | 4.2 |
|  |  | Post B | 126 | 126 | 100 | 97.1 | 100 | 5.4 | 4.5 | 6.4 |
| anti-4 | R1 | Post 3 | 141 | 140 | 99.3 | 96.1 | 100 | 3.5 | 3.0 | 4.0 |
|  |  | Post B | 132 | 132 | 100 | 97.2 | 100 | 6.1 | 5.1 | 7.2 |
|  | C1 | Post 3 | 134 | 134 | 100 | 97.3 | 100 | 4.2 | 3.5 | 4.9 |
|  |  | Post B | 125 | 125 | 100 | 97.1 | 100 | 6.8 | 5.7 | 8.0 |
| anti-5 | R1 | Post 3 | 141 | 141 | 100 | 97.4 | 100 | 5.1 | 4.5 | 5.8 |
|  |  | Post B | 133 | 132 | 99.2 | 95.9 | 100 | 6.5 | 5.5 | 7.8 |
|  | C1 | Post 3 | 135 | 135 | 100 | 97.3 | 100 | 6.5 | 5.6 | 7.4 |
|  |  | Post B | 126 | 126 | 100 | 97.1 | 100 | 7.6 | 6.4 | 9.1 |
| anti-6B | R1 | Post 3 | 141 | 123 | 87.2 | 80.6 | 92.3 | 1.1 | 0.8 | 1.3 |
|  |  | Post B | 133 | 132 | 99.2 | 95.9 | 100 | 4.7 | 4.0 | 5.5 |
|  | C1 | Post 3 | 135 | 118 | 87.4 | 80.6 | 92.5 | 1.2 | 1.0 | 1.6 |
|  |  | Post B | 126 | 125 | 99.2 | 95.7 | 100 | 4.1 | 3.5 | 4.9 |
| anti-7F | R1 | Post 3 | 141 | 141 | 100 | 97.4 | 100 | 4.4 | 3.9 | 4.9 |
|  |  | Post B | 133 | 133 | 100 | 97.3 | 100 | 7.1 | 6.2 | 8.2 |
|  | C1 | Post 3 | 135 | 135 | 100 | 97.3 | 100 | 4.9 | 4.3 | 5.7 |
|  |  | Post B | 126 | 126 | 100 | 97.1 | 100 | 7.2 | 6.3 | 8.2 |
| anti-9V | R1 | Post 3 | 141 | 137 | 97.2 | 92.9 | 99.2 | 2.8 | 2.4 | 3.3 |
|  |  | Post B | 133 | 133 | 100 | 97.3 | 100 | 6.0 | 5.1 | 7.1 |
|  | C1 | Post 3 | 135 | 134 | 99.3 | 95.9 | 100 | 3.7 | 3.3 | 4.2 |
|  |  | Post B | 126 | 126 | 100 | 97.1 | 100 | 5.7 | 4.9 | 6.6 |
| anti-14 | R1 | Post 3 | 141 | 141 | 100 | 97.4 | 100 | 5.8 | 5.0 | 6.7 |
|  |  | Post B | 133 | 132 | 99.2 | 95.9 | 100 | 9.0 | 7.6 | 10.7 |
|  | C1 | Post 3 | 134 | 132 | 98.5 | 94.7 | 99.8 | 5.7 | 4.7 | 7.0 |
|  |  | Post B | 126 | 126 | 100 | 97.1 | 100 | 9.0 | 7.4 | 10.8 |
| anti-18C | R1 | Post 3 | 141 | 139 | 98.6 | 95.0 | 99.8 | 3.4 | 2.8 | 4.1 |
|  |  | Post B | 133 | 133 | 100 | 97.3 | 100 | 13.7 | 11.5 | 16.3 |
|  | C1 | Post 3 | 134 | 134 | 100 | 97.3 | 100 | 6.2 | 5.1 | 7.5 |
|  |  | Post B | 126 | 126 | 100 | 97.1 | 100 | 14.5 | 12.3 | 17.2 |
| anti-19F | R1 | Post 3 | 141 | 139 | 98.6 | 95.0 | 99.8 | 4.2 | 3.4 | 5.2 |
|  |  | Post B | 133 | 132 | 99.2 | 95.9 | 100 | 6.0 | 4.9 | 7.4 |
|  | C1 | Post 3 | 134 | 129 | 96.3 | 91.5 | 98.8 | 5.1 | 4.1 | 6.4 |
|  |  | Post B | 126 | 126 | 100 | 97.1 | 100 | 7.2 | 5.8 | 8.8 |
| anti-23F | R1 | Post 3 | 140 | 129 | 92.1 | 86.4 | 96.0 | 1.3 | 1.1 | 1.6 |
|  |  | Post B | 133 | 131 | 98.5 | 94.7 | 99.8 | 4.1 | 3.4 | 5.1 |
|  | C1 | Post 3 | 134 | 120 | 89.6 | 83.1 | 94.2 | 1.5 | 1.1 | 1.9 |
|  |  | Post B | 126 | 124 | 98.4 | 94.4 | 99.8 | 3.9 | 3.2 | 4.8 |

Group R1 received RTS,S/AS01 + (DTPa/Hib + tOPV + PHiD-CV), and HRV 2 weeks later

Group C1 received HBV + (DTPa/Hib + tOPV + PHiD-CV), and HRV 2 weeks later

ATP = according to protocol; GMC = geometric mean antibody concentration calculated on all subjects; LL = lower limit; UL = upper limit; N = number of subjects with available results, n/% = number/percentage with titer ≥specified value; 95% CI = 95% confidence interval; Post 3 = one month post-dose 3, Post B = one month post booster

**Supplement table 4. Percentages of infants with serotype-specific pneumococcal opsonophagocytic activity (OPA) titers ≥8 and geometric mean OPA titers one month post-dose 3 and one month post-booster (Adapted ATP immunogenicity cohort).**

|  | | | | **≥ 8 (1/DIL)** | | | | **GMT** | | |
| --- | --- | --- | --- | --- | --- | --- | --- | --- | --- | --- |
|  | | | |  | | **95% CI** | |  | **95% CI** | |
| **Antibody** | **Group** | **Timing** | **N** | **n** | **%** | **LL** | **UL** | **value** | **LL** | **UL** |
| OPA-1 | R1 | Post 3 | 132 | 89 | 67.4 | 58.7 | 75.3 | 48.9 | 34.6 | 68.9 |
|  |  | Post B | 130 | 122 | 93.8 | 88.2 | 97.3 | 649.9 | 464.7 | 908.9 |
|  | C1 | Post 3 | 124 | 88 | 71.0 | 62.1 | 78.8 | 65.0 | 45.0 | 93.7 |
|  |  | Post B | 121 | 116 | 95.9 | 90.6 | 98.6 | 840.1 | 603.4 | 1169.7 |
| OPA-4 | R1 | Post 3 | 130 | 127 | 97.7 | 93.4 | 99.5 | 768.3 | 617.6 | 955.8 |
|  |  | Post B | 126 | 123 | 97.6 | 93.2 | 99.5 | 2347.1 | 1847.4 | 2982.0 |
|  | C1 | Post 3 | 123 | 123 | 100 | 97.0 | 100 | 810.9 | 676.5 | 972.0 |
|  |  | Post B | 116 | 115 | 99.1 | 95.3 | 100 | 2527.8 | 2064.1 | 3095.7 |
| OPA-5 | R1 | Post 3 | 133 | 126 | 94.7 | 89.5 | 97.9 | 77.6 | 61.9 | 97.3 |
|  |  | Post B | 129 | 124 | 96.1 | 91.2 | 98.7 | 324.2 | 244.1 | 430.5 |
|  | C1 | Post 3 | 124 | 116 | 93.5 | 87.7 | 97.2 | 93.8 | 73.6 | 119.6 |
|  |  | Post B | 121 | 117 | 96.7 | 91.8 | 99.1 | 392.8 | 291.3 | 529.6 |
| OPA-6B | R1 | Post 3 | 128 | 107 | 83.6 | 76.0 | 89.5 | 444.4 | 295.0 | 669.5 |
|  |  | Post B | 130 | 127 | 97.7 | 93.4 | 99.5 | 955.3 | 761.4 | 1198.6 |
|  | C1 | Post 3 | 121 | 98 | 81.0 | 72.9 | 87.6 | 389.3 | 250.1 | 606.1 |
|  |  | Post B | 116 | 114 | 98.3 | 93.9 | 99.8 | 828.2 | 652.7 | 1050.9 |
| OPA-7F | R1 | Post 3 | 132 | 132 | 100 | 97.2 | 100 | 3774.0 | 3232.7 | 4405.8 |
|  |  | Post B | 130 | 130 | 100 | 97.2 | 100 | 9167.3 | 7979.2 | 10532.3 |
|  | C1 | Post 3 | 124 | 124 | 100 | 97.1 | 100 | 3947.4 | 3338.3 | 4667.7 |
|  |  | Post B | 118 | 118 | 100 | 96.9 | 100 | 7794.6 | 6577.6 | 9236.8 |
| OPA-9V | R1 | Post 3 | 132 | 128 | 97.0 | 92.4 | 99.2 | 1257.7 | 977.3 | 1618.7 |
|  |  | Post B | 130 | 129 | 99.2 | 95.8 | 100 | 3035.3 | 2523.3 | 3651.3 |
|  | C1 | Post 3 | 122 | 121 | 99.2 | 95.5 | 100 | 1469.3 | 1180.4 | 1828.8 |
|  |  | Post B | 118 | 118 | 100 | 96.9 | 100 | 3164.6 | 2669.8 | 3751.1 |
| OPA-14 | R1 | Post 3 | 132 | 131 | 99.2 | 95.9 | 100 | 1426.3 | 1136.0 | 1790.9 |
|  |  | Post B | 127 | 126 | 99.2 | 95.7 | 100 | 1975.7 | 1565.8 | 2493.0 |
|  | C1 | Post 3 | 123 | 118 | 95.9 | 90.8 | 98.7 | 1269.0 | 965.1 | 1668.6 |
|  |  | Post B | 119 | 117 | 98.3 | 94.1 | 99.8 | 1865.0 | 1463.9 | 2375.9 |
| OPA-18C | R1 | Post 3 | 124 | 110 | 88.7 | 81.8 | 93.7 | 192.6 | 139.2 | 266.4 |
|  |  | Post B | 127 | 123 | 96.9 | 92.1 | 99.1 | 1694.1 | 1188.6 | 2414.7 |
|  | C1 | Post 3 | 118 | 109 | 92.4 | 86.0 | 96.5 | 249.7 | 185.0 | 337.0 |
|  |  | Post B | 115 | 112 | 97.4 | 92.6 | 99.5 | 1548.7 | 1096.3 | 2188.0 |
| OPA-19F | R1 | Post 3 | 129 | 105 | 81.4 | 73.6 | 87.7 | 159.3 | 109.9 | 231.0 |
|  |  | Post B | 130 | 112 | 86.2 | 79.0 | 91.6 | 344.5 | 223.0 | 532.3 |
|  | C1 | Post 3 | 123 | 106 | 86.2 | 78.8 | 91.7 | 228.8 | 160.4 | 326.3 |
|  |  | Post B | 121 | 110 | 90.9 | 84.3 | 95.4 | 469.7 | 320.0 | 689.4 |
| OPA-23F | R1 | Post 3 | 132 | 109 | 82.6 | 75.0 | 88.6 | 760.9 | 476.3 | 1215.5 |
|  |  | Post B | 127 | 125 | 98.4 | 94.4 | 99.8 | 3199.8 | 2543.7 | 4025.1 |
|  | C1 | Post 3 | 121 | 99 | 81.8 | 73.8 | 88.2 | 735.6 | 456.3 | 1185.9 |
|  |  | Post B | 118 | 117 | 99.2 | 95.4 | 100 | 3198.1 | 2526.5 | 4048.4 |

Group R1 received RTS,S/AS01 + (DTPa/Hib + tOPV + PHiD-CV), and HRV 2 weeks later

Group C1 received HBV + (DTPa/Hib + tOPV + PHiD-CV), and HRV 2 weeks later

ATP = According to protocol; GMT = geometric mean antibody titer calculated on all subjects

N = number of subjects with available results, n/% = number/percentage with titer ≥ specified value;

95% CI = 95% confidence interval; Post 3 = one month post-dose 3, Post B = one month post booster

**Supplement Table 5. Percentages of infants with Protein D antibody concentrations ≥100 EU/ml and geometric mean antibody concentrations one month after primary vaccination with PHiD-CV co-administered with RTS,S/AS01 or HBV (ATP cohort for immunogenicity)**

|  | | **≥ 100 EU/ml** | | | **GMC** | |
| --- | --- | --- | --- | --- | --- | --- |
| **Group** | **N** | **n** | **%** | **(95% CI)** | **value** | **(95% CI)** |
| R1 | 141 | 141 | 100 | (97.4; 100) | 2435.3 | (2204.3; 2690.6) |
| C1 | 134 | 134 | 100 | (97.3; 100) | 2956.7 | (2647.5; 3302.1) |

Group R1 received RTS,S/AS01 + (DTPa/Hib + tOPV + PHiD-CV), and HRV 2 weeks later

Group C1 received HBV + (DTPa/Hib + tOPV + PHiD-CV), and HRV 2 weeks later

ATP = According to protocol; GMC = geometric mean antibody concentration calculated on all subjects

N = number of subjects with available results

n/% = number/percentage of subjects with titer equal to or above specified value

95% CI = 95% confidence interval; LL = Lower Limit, UL = Upper Limit

PIII(M3) = Post Dose 3, Month 3

**Supplement Table 6. Pertussis antibody seropositivity, geometric mean concentrations (GMCs) and vaccine response (VR) rates one month post-dose 3 (ATP immunogenicity cohort)**

|  | | | | **≥ 5 EU/ml** | | | **GMC** | | **VR** | | | |
| --- | --- | --- | --- | --- | --- | --- | --- | --- | --- | --- | --- | --- |
| **Antigen** | **Group** | **Timing** | **N** | **n** | **%** | **(95% CI)** | **value** | **(95% CI)** | **N** | **n** | **%** | **(95% CI)** |
| Pertussis toxin | R | Screening | 401 | 116 | 28.9 | (24.5; 33.6) | 3.8 | (3.6; 4.1) | - | - | - | - |
|  |  | Post III | 387 | 387 | 100 | (99.1; 100) | 105.9 | (99.2; 113.1) | 386 | 383 | 99.2 | (97.7; 99.8) |
|  | C | Screening | 253 | 90 | 35.6 | (29.7; 41.8) | 4.3 | (3.9; 4.8) | - | - | - | - |
|  |  | Post III | 247 | 247 | 100 | (98.5; 100) | 114.2 | (104.8; 124.5) | 246 | 241 | 98.0 | (95.3; 99.3) |
| Filamentous haemagglutinin | R | Screening | 399 | 359 | 90.0 | (86.6; 92.7) | 13.9 | (12.7; 15.2) | - | - | - | - |
|  |  | Post III | 386 | 386 | 100 | (99.0; 100) | 271.1 | (252.8; 290.8) | 383 | 381 | 99.5 | (98.1; 99.9) |
|  | C | Screening | 253 | 237 | 93.7 | (89.9; 96.3) | 15.7 | (14.1; 17.5) | - | - | - | - |
|  |  | Post III | 247 | 247 | 100 | (98.5; 100) | 292.9 | (268.9; 319.1) | 246 | 243 | 98.8 | (96.5; 99.7) |
| Pertactin | R | Screening | 401 | 70 | 17.5 | (13.9; 21.5) | 3.2 | (3.0; 3.4) | - | - | - | - |
|  |  | Post III | 387 | 387 | 100 | (99.1; 100) | 164.1 | (153.6; 175.3) | 386 | 385 | 99.7 | (98.6; 100) |
|  | C | Screening | 253 | 48 | 19.0 | (14.3; 24.4) | 3.2 | (3.0; 3.5) | - | - | - | - |
|  |  | Post III | 247 | 247 | 100 | (98.5; 100) | 179.7 | (164.4; 196.5) | 246 | 246 | 100 | (98.5; 100) |

Group R = All study groups that received RTS,S/AS01 vaccine.

Group C = All study groups that received HBV vaccine.

VR (vaccine response) seroconversion in initially seronegative infants or an antibody titer post-vaccination at least equal to the titer prior to vaccination in initially seropositive infants

GMC calculated on all subjects,

N = number of subjects with available results (for VR, N = number of subjects with pre- and post-vaccination results available),

n/% = number/percentage of subjects with titer ≥ specified value,

95% CI = 95% confidence interval,

Screening = Pre-vaccination, Post III = one month post-dose 3.

**Supplement Table 7. Solicited local symptoms at any injection site over 7 days post primary vaccination (Days 0-6) by dose and overall (Total vaccinated cohort)**

|  | | **R1** | | | | **R2** | | | | **R3** | | | | **C1** | | | | **C2** | | | |
| --- | --- | --- | --- | --- | --- | --- | --- | --- | --- | --- | --- | --- | --- | --- | --- | --- | --- | --- | --- | --- | --- |
| **Symptom** | **Type** | **N** | **n** | **%** | **(95 % CI)** | **N** | **n** | **%** | **(95 % CI)** | **N** | **n** | **%** | **(95 % CI)** | **N** | **n** | **%** | **(95 % CI)** | **N** | **n** | **%** | **(95 % CI)** |
| **Dose 1** | | | | | | | | | | | | | | | | | | | | | |
| Pain | All | 142 | 41 | 28.9 | (21.6; 37.1) | 142 | 28 | 19.7 | (13.5; 27.2) | 141 | 31 | 22.0 | (15.5; 29.7) | 141 | 29 | 20.6 | (14.2; 28.2) | 139 | 15 | 10.8 | (6.2; 17.2) |
|  | Grade 3 | 142 | 0 | 0.0 | (0.0; 2.6) | 142 | 0 | 0.0 | (0.0; 2.6) | 141 | 1 | 0.7 | (0.0; 3.9) | 141 | 0 | 0.0 | (0.0; 2.6) | 139 | 0 | 0.0 | (0.0; 2.6) |
| Redness (mm) | All | 142 | 1 | 0.7 | (0.0; 3.9) | 142 | 0 | 0.0 | (0.0; 2.6) | 141 | 2 | 1.4 | (0.2; 5.0) | 141 | 5 | 3.5 | (1.2; 8.1) | 139 | 1 | 0.7 | (0.0; 3.9) |
|  | >20.0 | 142 | 0 | 0.0 | (0.0; 2.6) | 142 | 0 | 0.0 | (0.0; 2.6) | 141 | 0 | 0.0 | (0.0; 2.6) | 141 | 0 | 0.0 | (0.0; 2.6) | 139 | 0 | 0.0 | (0.0; 2.6) |
| Swelling (mm) | All | 142 | 5 | 3.5 | (1.2; 8.0) | 142 | 2 | 1.4 | (0.2; 5.0) | 141 | 6 | 4.3 | (1.6; 9.0) | 141 | 10 | 7.1 | (3.5; 12.7) | 139 | 4 | 2.9 | (0.8; 7.2) |
|  | >20.0 | 142 | 0 | 0.0 | (0.0; 2.6) | 142 | 0 | 0.0 | (0.0; 2.6) | 141 | 0 | 0.0 | (0.0; 2.6) | 141 | 0 | 0.0 | (0.0; 2.6) | 139 | 0 | 0.0 | (0.0; 2.6) |
| **Dose 2** | | | | | | | | | | | | | | | | | | | | | |
| Pain | All | 141 | 30 | 21.3 | (14.8; 29.0) | 126 | 14 | 11.1 | (6.2; 17.9) | 141 | 21 | 14.9 | (9.5; 21.9) | 141 | 24 | 17.0 | (11.2; 24.3) | 123 | 9 | 7.3 | (3.4; 13.4) |
|  | Grade 3 | 141 | 1 | 0.7 | (0.0; 3.9) | 126 | 0 | 0.0 | (0.0; 2.9) | 141 | 0 | 0.0 | (0.0; 2.6) | 141 | 0 | 0.0 | (0.0; 2.6) | 123 | 0 | 0.0 | (0.0; 3.0) |
| Redness (mm) | All | 141 | 5 | 3.5 | (1.2; 8.1) | 126 | 1 | 0.8 | (0.0; 4.3) | 141 | 2 | 1.4 | (0.2; 5.0) | 141 | 3 | 2.1 | (0.4; 6.1) | 123 | 0 | 0.0 | (0.0; 3.0) |
|  | >20.0 | 141 | 0 | 0.0 | (0.0; 2.6) | 126 | 0 | 0.0 | (0.0; 2.9) | 141 | 0 | 0.0 | (0.0; 2.6) | 141 | 0 | 0.0 | (0.0; 2.6) | 123 | 0 | 0.0 | (0.0; 3.0) |
| Swelling (mm) | All | 141 | 8 | 5.7 | (2.5; 10.9) | 126 | 3 | 2.4 | (0.5; 6.8) | 141 | 4 | 2.8 | (0.8; 7.1) | 141 | 9 | 6.4 | (3.0; 11.8) | 123 | 4 | 3.3 | (0.9; 8.1) |
|  | >20.0 | 141 | 0 | 0.0 | (0.0; 2.6) | 126 | 0 | 0.0 | (0.0; 2.9) | 141 | 0 | 0.0 | (0.0; 2.6) | 141 | 0 | 0.0 | (0.0; 2.6) | 123 | 0 | 0.0 | (0.0; 3.0) |
| **Dose 3** | | | | | | | | | | | | | | | | | | | | | |
| Pain | All | 141 | 14 | 9.9 | (5.5; 16.1) | 126 | 10 | 7.9 | (3.9; 14.1) | 141 | 14 | 9.9 | (5.5; 16.1) | 141 | 18 | 12.8 | (7.7; 19.4) | 123 | 7 | 5.7 | (2.3; 11.4) |
|  | Grade 3 | 141 | 0 | 0.0 | (0.0; 2.6) | 126 | 0 | 0.0 | (0.0; 2.9) | 141 | 0 | 0.0 | (0.0; 2.6) | 141 | 0 | 0.0 | (0.0; 2.6) | 123 | 0 | 0.0 | (0.0; 3.0) |
| Redness (mm) | All | 141 | 3 | 2.1 | (0.4; 6.1) | 126 | 0 | 0.0 | (0.0; 2.9) | 141 | 1 | 0.7 | (0.0; 3.9) | 141 | 3 | 2.1 | (0.4; 6.1) | 123 | 0 | 0.0 | (0.0; 3.0) |
|  | >20.0 | 141 | 0 | 0.0 | (0.0; 2.6) | 126 | 0 | 0.0 | (0.0; 2.9) | 141 | 0 | 0.0 | (0.0; 2.6) | 141 | 0 | 0.0 | (0.0; 2.6) | 123 | 0 | 0.0 | (0.0; 3.0) |
| Swelling (mm) | All | 141 | 7 | 5.0 | (2.0; 10.0) | 126 | 2 | 1.6 | (0.2; 5.6) | 141 | 6 | 4.3 | (1.6; 9.0) | 141 | 11 | 7.8 | (4.0; 13.5) | 123 | 3 | 2.4 | (0.5; 7.0) |
|  | >20.0 | 141 | 0 | 0.0 | (0.0; 2.6) | 126 | 0 | 0.0 | (0.0; 2.9) | 141 | 0 | 0.0 | (0.0; 2.6) | 141 | 0 | 0.0 | (0.0; 2.6) | 123 | 0 | 0.0 | (0.0; 3.0) |
| **Overall/dose** | | | | | | | | | | | | | | | | | | | | | |
| Pain | All | 424 | 85 | 20.0 | (16.3; 24.2) | 394 | 52 | 13.2 | (10.0; 16.9) | 423 | 66 | 15.6 | (12.3; 19.4) | 423 | 71 | 16.8 | (13.3; 20.7) | 385 | 31 | 8.1 | (5.5; 11.2) |
|  | Grade 3 | 424 | 1 | 0.2 | (0.0; 1.3) | 394 | 0 | 0.0 | (0.0; 0.9) | 423 | 1 | 0.2 | (0.0; 1.3) | 423 | 0 | 0.0 | (0.0; 0.9) | 385 | 0 | 0.0 | (0.0; 1.0) |
| Redness (mm) | All | 424 | 9 | 2.1 | (1.0; 4.0) | 394 | 1 | 0.3 | (0.0; 1.4) | 423 | 5 | 1.2 | (0.4; 2.7) | 423 | 11 | 2.6 | (1.3; 4.6) | 385 | 1 | 0.3 | (0.0; 1.4) |
|  | >20.0 | 424 | 0 | 0.0 | (0.0; 0.9) | 394 | 0 | 0.0 | (0.0; 0.9) | 423 | 0 | 0.0 | (0.0; 0.9) | 423 | 0 | 0.0 | (0.0; 0.9) | 385 | 0 | 0.0 | (0.0; 1.0) |
| Swelling (mm) | All | 424 | 20 | 4.7 | (2.9; 7.2) | 394 | 7 | 1.8 | (0.7; 3.6) | 423 | 16 | 3.8 | (2.2; 6.1) | 423 | 30 | 7.1 | (4.8; 10.0) | 385 | 11 | 2.9 | (1.4; 5.1) |
|  | >20.0 | 424 | 0 | 0.0 | (0.0; 0.9) | 394 | 0 | 0.0 | (0.0; 0.9) | 423 | 0 | 0.0 | (0.0; 0.9) | 423 | 0 | 0.0 | (0.0; 0.9) | 385 | 0 | 0.0 | (0.0; 1.0) |

Group R1 received RTS,S/AS01 + (DTPa/Hib + tOPV + PHiD-CV), and HRV 2 weeks later

Group R2 received RTS,S/AS01 + (DTPa/Hib + tOPV + HRV), and PHiD-CV 2 weeks later

Group R3 received RTS,S/AS01 + (DTPa/Hib + tOPV), and (PHiD-CV + HRV) 2 weeks later

Group C1 received HBV + (DTPa/Hib + tOPV + PHiD-CV), and HRV 2 weeks later

Group C2 received HBV + (DTPa/Hib + tOPV + HRV), and PHiD-CV 2 weeks later

For each dose N = number of subjects with at least one administered dose, n/% = number/percentage reporting the symptom at least once,

For Overall/dose: N = number of administered doses, n/% = number/percentage of doses followed by at least one type of symptom, 95%CI = Exact 95% confidence interval

Grade 3: Pain = Cries when limb is moved/spontaneously painful.

**Supplement Table 8. Solicited general symptoms over 7 days post primary vaccination (Days 0-6) by dose and overall (Total vaccinated cohort)**

|  | | **R1** | | | | **R2** | | | | **R3** | | | | **C1** | | | | **C2** | | | |
| --- | --- | --- | --- | --- | --- | --- | --- | --- | --- | --- | --- | --- | --- | --- | --- | --- | --- | --- | --- | --- | --- |
| **Symptom** | **Intensity** | **N** | **n** | **%** | **(95 % CI)** | **N** | **n** | **%** | **(95 % CI)** | **N** | **n** | **%** | **(95 % CI)** | **N** | **n** | **%** | **(95 % CI)** | **N** | **n** | **%** | **(95 % CI)** |
| **Dose 1** | | | | | | | | | | | | | | | | | | | | | |
| Drowsiness | All | 142 | 2 | 1.4 | (0.2; 5.0) | 142 | 1 | 0.7 | (0.0; 3.9) | 141 | 3 | 2.1 | (0.4; 6.1) | 141 | 3 | 2.1 | (0.4; 6.1) | 139 | 0 | 0.0 | (0.0; 2.6) |
|  | Grade 3 | 142 | 0 | 0.0 | (0.0; 2.6) | 142 | 0 | 0.0 | (0.0; 2.6) | 141 | 0 | 0.0 | (0.0; 2.6) | 141 | 0 | 0.0 | (0.0; 2.6) | 139 | 0 | 0.0 | (0.0; 2.6) |
| Irritability/fussiness | All | 142 | 15 | 10.6 | (6.0; 16.8) | 142 | 11 | 7.7 | (3.9; 13.4) | 141 | 11 | 7.8 | (4.0; 13.5) | 141 | 9 | 6.4 | (3.0; 11.8) | 139 | 5 | 3.6 | (1.2; 8.2) |
|  | Grade 3 | 142 | 0 | 0.0 | (0.0; 2.6) | 142 | 0 | 0.0 | (0.0; 2.6) | 141 | 0 | 0.0 | (0.0; 2.6) | 141 | 0 | 0.0 | (0.0; 2.6) | 139 | 0 | 0.0 | (0.0; 2.6) |
| Loss of appetite | All | 142 | 4 | 2.8 | (0.8; 7.1) | 142 | 1 | 0.7 | (0.0; 3.9) | 141 | 2 | 1.4 | (0.2; 5.0) | 141 | 4 | 2.8 | (0.8; 7.1) | 139 | 0 | 0.0 | (0.0; 2.6) |
|  | Grade 3 | 142 | 0 | 0.0 | (0.0; 2.6) | 142 | 0 | 0.0 | (0.0; 2.6) | 141 | 0 | 0.0 | (0.0; 2.6) | 141 | 0 | 0.0 | (0.0; 2.6) | 139 | 0 | 0.0 | (0.0; 2.6) |
| Fever | ≥37.5°C | 142 | 44 | 31.0 | (23.5; 39.3) | 142 | 20 | 14.1 | (8.8; 20.9) | 141 | 16 | 11.3 | (6.6; 17.8) | 141 | 23 | 16.3 | (10.6; 23.5) | 139 | 13 | 9.4 | (5.1; 15.5) |
|  | >39.0°C | 142 | 0 | 0.0 | (0.0; 2.6) | 142 | 0 | 0.0 | (0.0; 2.6) | 141 | 0 | 0.0 | (0.0; 2.6) | 141 | 0 | 0.0 | (0.0; 2.6) | 139 | 0 | 0.0 | (0.0; 2.6) |
| **Dose 2** | | | | | | | | | | | | | | | | | | | | | |
| Drowsiness | All | 141 | 5 | 3.5 | (1.2; 8.1) | 126 | 1 | 0.8 | (0.0; 4.3) | 141 | 1 | 0.7 | (0.0; 3.9) | 141 | 3 | 2.1 | (0.4; 6.1) | 123 | 0 | 0.0 | (0.0; 3.0) |
|  | Grade 3 | 141 | 0 | 0.0 | (0.0; 2.6) | 126 | 0 | 0.0 | (0.0; 2.9) | 141 | 0 | 0.0 | (0.0; 2.6) | 141 | 0 | 0.0 | (0.0; 2.6) | 123 | 0 | 0.0 | (0.0; 3.0) |
| Irritability/fussiness | All | 141 | 13 | 9.2 | (5.0; 15.3) | 126 | 7 | 5.6 | (2.3; 11.1) | 141 | 12 | 8.5 | (4.5; 14.4) | 141 | 10 | 7.1 | (3.5; 12.7) | 123 | 0 | 0.0 | (0.0; 3.0) |
|  | Grade 3 | 141 | 0 | 0.0 | (0.0; 2.6) | 126 | 0 | 0.0 | (0.0; 2.9) | 141 | 0 | 0.0 | (0.0; 2.6) | 141 | 0 | 0.0 | (0.0; 2.6) | 123 | 0 | 0.0 | (0.0; 3.0) |
| Loss of appetite | All | 141 | 3 | 2.1 | (0.4; 6.1) | 126 | 1 | 0.8 | (0.0; 4.3) | 141 | 1 | 0.7 | (0.0; 3.9) | 141 | 3 | 2.1 | (0.4; 6.1) | 123 | 0 | 0.0 | (0.0; 3.0) |
|  | Grade 3 | 141 | 0 | 0.0 | (0.0; 2.6) | 126 | 0 | 0.0 | (0.0; 2.9) | 141 | 0 | 0.0 | (0.0; 2.6) | 141 | 0 | 0.0 | (0.0; 2.6) | 123 | 0 | 0.0 | (0.0; 3.0) |
| Fever | ≥37.5°C | 141 | 30 | 21.3 | (14.8; 29.0) | 126 | 14 | 11.1 | (6.2; 17.9) | 141 | 18 | 12.8 | (7.7; 19.4) | 141 | 20 | 14.2 | (8.9; 21.1) | 123 | 5 | 4.1 | (1.3; 9.2) |
|  | >39.0°C | 141 | 2 | 1.4 | (0.2; 5.0) | 126 | 0 | 0.0 | (0.0; 2.9) | 141 | 1 | 0.7 | (0.0; 3.9) | 141 | 1 | 0.7 | (0.0; 3.9) | 123 | 0 | 0.0 | (0.0; 3.0) |
| **Dose 3** | | | | | | | | | | | | | | | | | | | | | |
| Drowsiness | All | 141 | 3 | 2.1 | (0.4; 6.1) | 126 | 0 | 0.0 | (0.0; 2.9) | 141 | 2 | 1.4 | (0.2; 5.0) | 141 | 1 | 0.7 | (0.0; 3.9) | 123 | 0 | 0.0 | (0.0; 3.0) |
|  | Grade 3 | 141 | 0 | 0.0 | (0.0; 2.6) | 126 | 0 | 0.0 | (0.0; 2.9) | 141 | 0 | 0.0 | (0.0; 2.6) | 141 | 0 | 0.0 | (0.0; 2.6) | 123 | 0 | 0.0 | (0.0; 3.0) |
| Irritability/fussiness | All | 141 | 5 | 3.5 | (1.2; 8.1) | 126 | 3 | 2.4 | (0.5; 6.8) | 141 | 10 | 7.1 | (3.5; 12.7) | 141 | 6 | 4.3 | (1.6; 9.0) | 123 | 1 | 0.8 | (0.0; 4.4) |
|  | Grade 3 | 141 | 0 | 0.0 | (0.0; 2.6) | 126 | 0 | 0.0 | (0.0; 2.9) | 141 | 0 | 0.0 | (0.0; 2.6) | 141 | 0 | 0.0 | (0.0; 2.6) | 123 | 0 | 0.0 | (0.0; 3.0) |
| Loss of appetite | All | 141 | 2 | 1.4 | (0.2; 5.0) | 126 | 0 | 0.0 | (0.0; 2.9) | 141 | 1 | 0.7 | (0.0; 3.9) | 141 | 1 | 0.7 | (0.0; 3.9) | 123 | 1 | 0.8 | (0.0; 4.4) |
|  | Grade 3 | 141 | 0 | 0.0 | (0.0; 2.6) | 126 | 0 | 0.0 | (0.0; 2.9) | 141 | 0 | 0.0 | (0.0; 2.6) | 141 | 0 | 0.0 | (0.0; 2.6) | 123 | 0 | 0.0 | (0.0; 3.0) |
| Fever | ≥37.5°C | 141 | 38 | 27.0 | (19.8; 35.1) | 126 | 20 | 15.9 | (10.0; 23.4) | 141 | 26 | 18.4 | (12.4; 25.8) | 141 | 16 | 11.3 | (6.6; 17.8) | 123 | 12 | 9.8 | (5.1; 16.4) |
|  | >39.0°C | 141 | 3 | 2.1 | (0.4; 6.1) | 126 | 0 | 0.0 | (0.0; 2.9) | 141 | 2 | 1.4 | (0.2; 5.0) | 141 | 0 | 0.0 | (0.0; 2.6) | 123 | 4 | 3.3 | (0.9; 8.1) |
| **Overall/dose** | | | | | | | | | | | | | | | | | | | | | |
| Drowsiness | All | 424 | 10 | 2.4 | (1.1; 4.3) | 394 | 2 | 0.5 | (0.1; 1.8) | 423 | 6 | 1.4 | (0.5; 3.1) | 423 | 7 | 1.7 | (0.7; 3.4) | 385 | 0 | 0.0 | (0.0; 1.0) |
|  | Grade 3 | 424 | 0 | 0.0 | (0.0; 0.9) | 394 | 0 | 0.0 | (0.0; 0.9) | 423 | 0 | 0.0 | (0.0; 0.9) | 423 | 0 | 0.0 | (0.0; 0.9) | 385 | 0 | 0.0 | (0.0; 1.0) |
| Irritability/fussiness | All | 424 | 33 | 7.8 | (5.4; 10.8) | 394 | 21 | 5.3 | (3.3; 8.0) | 423 | 33 | 7.8 | (5.4; 10.8) | 423 | 25 | 5.9 | (3.9; 8.6) | 385 | 6 | 1.6 | (0.6; 3.4) |
|  | Grade 3 | 424 | 0 | 0.0 | (0.0; 0.9) | 394 | 0 | 0.0 | (0.0; 0.9) | 423 | 0 | 0.0 | (0.0; 0.9) | 423 | 0 | 0.0 | (0.0; 0.9) | 385 | 0 | 0.0 | (0.0; 1.0) |
| Loss of appetite | All | 424 | 9 | 2.1 | (1.0; 4.0) | 394 | 2 | 0.5 | (0.1; 1.8) | 423 | 4 | 0.9 | (0.3; 2.4) | 423 | 8 | 1.9 | (0.8; 3.7) | 385 | 1 | 0.3 | (0.0; 1.4) |
|  | Grade 3 | 424 | 0 | 0.0 | (0.0; 0.9) | 394 | 0 | 0.0 | (0.0; 0.9) | 423 | 0 | 0.0 | (0.0; 0.9) | 423 | 0 | 0.0 | (0.0; 0.9) | 385 | 0 | 0.0 | (0.0; 1.0) |
| Fever | ≥37.5°C | 424 | 112 | 26.4 | (22.3; 30.9) | 394 | 54 | 13.7 | (10.5; 17.5) | 423 | 60 | 14.2 | (11.0; 17.9) | 423 | 59 | 13.9 | (10.8; 17.6) | 385 | 30 | 7.8 | (5.3; 10.9) |
|  | >39.0°C | 424 | 5 | 1.2 | (0.4; 2.7) | 394 | 0 | 0.0 | (0.0; 0.9) | 423 | 3 | 0.7 | (0.1; 2.1) | 423 | 1 | 0.2 | (0.0; 1.3) | 385 | 4 | 1.0 | (0.3; 2.6) |

Group R1 received RTS,S/AS01 + (DTPa/Hib + tOPV + PHiD-CV), and HRV 2 weeks later

Group R2 received RTS,S/AS01 + (DTPa/Hib + tOPV + HRV), and PHiD-CV 2 weeks later

Group R3 received RTS,S/AS01 + (DTPa/Hib + tOPV), and (PHiD-CV + HRV) 2 weeks later

Group C1 received HBV + (DTPa/Hib + tOPV + PHiD-CV), and HRV 2 weeks later

Group C2 received HBV + (DTPa/Hib + tOPV + HRV), and PHiD-CV 2 weeks later

For each dose N = number of subjects with at least one administered dose, n/% = number/percentage of subjects reporting the symptom at least once, For Overall/dose: N = number of administered doses, n/% = number/percentage of doses followed by at least one type of symptom, 95%CI = Exact 95% confidence interval. Temperature (axillary route).

Grade 3: Irritability/Fussiness = Crying that cannot be comforted/prevents normal activity, Drowsiness = Drowsiness that prevents normal activity, Loss of appetite = Not eating at all

*The analysis of safety was done considering the treatment actually administered

**Supplement 9**

**Study oversight and ethical considerations**

**Informed consent of pregnant women**

Pregnant women were identified at antenatal clinics or in the community and were informed about the study. If interested in participating they were asked to sign a consent form for screening for hepatitis B or human immunodeficiency virus (HIV) infection. Mothers who were screened negative for hepatitis B surface antigen (HBsAg) and HIV and who agreed to let their child participate in the study were invited for the screening visit (Visit 1).

**Informed consent for infants**

Before performing any other study procedure, the signed/thumb-printed informed consent of the subject’s parent(s)/Legally acceptable representative (LAR) was obtained. When the parent(s)/LAR(s) of the child were illiterate, the informed consent form (ICF) was also signed and dated by a literate impartial witness.

**Independent Data Monitoring Committee (IDMC) oversight**

This study was overseen by an IDMC operating under a charter and assisted by a local safety monitor. The IDMC reviewed safety data and authorized trial continuation.

### Screening activities, inclusion and exclusion criteria

**Screening of pregnant women**

- Pregnant women were identified at antenatal clinics or in the community and were informed about the study. If interested in participating they were asked to sign a consent form for screening for hepatitis B or HIV infection.
- Infants born to mothers who were HBsAg positive and therefore at high risk of vertical transmission of hepatitis B infection were excluded from this trial. These infants were offered a licensed hepatitis B vaccine in a schedule beginning at birth. Women who tested positive for HBsAg with symptoms were referred to an appropriate health care center.
- Infants born to mothers who were HIV positive were excluded from this trial. Voluntary counselling and testing, highly active anti-retroviral therapy (HAART) and prevention of mother-to-child transmission (PMCT) are available at the study center according to national policies.
- The site study team ensured that insecticide treated bednet use is optimized in the study population.

**Screening of infants**

- Mothers who were screened negative for HBsAg and HIV and who agreed to let their child participate in the study were invited for the screening visit (Visit 1).
- Prior to any other study procedures, the parents were asked to sign/thumb-print a consent form to allow their child to participate in the trial.

### Inclusion criteria

*All subjects had to satisfy ALL the following criteria at study entry:*

- A male or female infant aged between 8 and 12 weeks inclusive at the time of first vaccination.
- Signed or thumb-printed informed consent obtained from the parent(s)/LAR(s) of the child. Where parent(s)/LAR(s) were illiterate, the consent form was countersigned by an independent witness.
- Subjects who the investigator believed that their parent(s)/LAR(s) could and would comply with the requirements of the protocol (e.g. return for follow-up visits).
- Healthy subjects as established by medical history and clinical examination before entering into the study.
- Born to a mother who was HBsAg negative.
- Born to a mother who was HIV negative.
- Born after a normal gestation period of 36 to 42 weeks inclusive.

### Exclusion criteria

*The following criteria were checked at the time of study entry. If ANY exclusion criterion applied, the subject was not included in the study:*

- Child in care. Acute disease and/or fever at the time of enrolment.
- Fever was defined as temperature ≥ 37.5°C on oral, axillary or tympanic setting, or ≥ 38.0°C on rectal setting.
- Subjects with a minor illness (such as mild diarrhea, mild upper respiratory infection) without fever may have been enrolled at the discretion of the investigator.
- Serious acute or chronic illness determined by clinical or physical examination and laboratory screening tests including, but not limited to:
- Any confirmed or suspected immunosuppressive or immunodeficient condition, based on medical history and physical examination (no laboratory testing required).
- A family history of congenital or hereditary immunodeficiency.
- Major congenital defects.
- History of any neurologic disorders or seizures.
- Moderate malnutrition at screening defined as weight for age Z-score (WAZ) less than ‑2.
- Laboratory screening tests out of range, specifically:
- Alanine aminotransferase (ALT) above acceptable limit (i.e. > 60 IU/l).
- Creatinine above acceptable limit (i.e. > 60 µmol/l).
- Hemoglobin below acceptable limit (i.e. < 8.0 g/dl).
- Platelet count below acceptable limit (i.e. < 100 x 10^3^/µl).
- Total white cell count below acceptable limit (i.e. < 4.0 x 10^3^/µl).

Note that screening laboratory tests had to be performed within 14 days of Dose 1.

- Previous vaccination with diphtheria, tetanus, *B. pertussis* (whole-cell or acellular), *Haemophilus influenzae* type b, *Streptococcus pneumoniae*, hepatitis B vaccine or rotavirus vaccines.
- Planned administration/administration of a licensed vaccine (i.e. a vaccine that was approved by one of the following authorities: Food and Drug Administration [FDA] or European Union [EU] member state or WHO [with respect to prequalification]) not foreseen by the study protocol within 7 days of the first dose of study vaccine.
- Use of a drug or vaccine that was not approved for that indication (by one of the following authorities: FDA or EU member state or WHO [with respect to prequalification]) other than the study vaccines within 30 days preceding the first dose of study vaccine, or planned use during the study period.
- Administration of immunoglobulins and/or any blood products in the period between birth and Dose 1 and within the three months preceding planned vaccine administration during the study period.
- Chronic administration (defined as more than 14 days in total) of immuno-suppressants or other immune-modifying drugs in the period between birth and Dose 1. For corticosteroids, this meant prednisone ≥ 0.5 mg/kg/day, or equivalent. Inhaled and topical steroids were allowed.
- Concurrently participating in another clinical study at any time during the study period, in which the subject had been or would have been exposed to an investigational or a non-investigational product (pharmaceutical product or device).
- Same sex twin (to avoid misidentification).
- Maternal death.
- History of allergic reactions (significant IgE-mediated events) or anaphylaxis to previous immunizations.
- History of allergic disease or reactions likely to be exacerbated by any component of the vaccine.
- Any other findings that the investigator felt would have increased the risk of having an adverse outcome from participation in the trial.
- Any other findings that the investigator felt would have resulted in data collected being incomplete or of poor quality.
- Previous participation in any other malaria vaccine trial.

**Composition and administration route of study vaccines**

| **Vaccine** | **Composition** | **Dose volume** | **Route** |
| --- | --- | --- | --- |
| RTS,S/AS01 | 25 µg RTS,S antigen reconstituted with AS01 adjuvant, composed of *Quillaja saponaria* Molina, fraction 21 (QS-21) (25 micrograms) and 3-O-desacyl-4’- monophosphoryl lipid A (MPL) (25 micrograms) with liposomes | 0.5 ml | IM |
| *Engerix-B* | 10 μg of hepatitis B surface antigen adsorbed onto aluminium hydroxide. | 0.5 ml | IM |
| *Infanrix/Hib* | One dose (0.5 ml) ≥30 IU diphtheria toxoid, ≥40 IU tetanus toxoid, 25µg pertussis toxin, 25µg filamentous haemagglutinin, 8µg pertactin reconstituted with 10µg *Haemophilus influenzae* type b polysaccharide conjugated to tetanus toxoid. The diphtheria toxoid, tetanus toxoid and acellular *B. pertussis* vaccine components are adsorbed on aluminium salts. | 0.5 ml | IM |
| *Polio Sabin* | Stabilized preparation of live attenuated poliomyelitis viruses of Sabin strains with not less than 10^6^ CCID_50_ of Type 1 (strain LSc, 2ab), 10^5^ CCID_50_ of Type 2 (strain P 712 ch, and 2ab), 10^5.8^ CCID_50_ of Type 3 (strain Leon 12a, 1b). The viruses are propagated in human diploid cells. | 0.1 ml | Oral |
| *Synflorix* | Each 0.5 ml dose contains 1 μg of each capsular polysaccharide of serotypes 1, 5, 6B, 7F, 9V, 14 and 23F, and 3 μg of serotype 4, conjugated individually to Protein D; 3μg of capsular polysaccharide of serotype 18C conjugated to tetanus toxoid; and 3 μg of capsular polysaccharide of serotype 19F conjugated to diphtheria toxoid., adsorbed on aluminium phosphate | 0.5 ml | IM |
| *Rotarix* | Not less than 10^6.0^ CCID_50_ of live attenuated human rotavirus RIX4414 strain. | 1.0 ml | Oral |

CCID50 = Cell Culture Infectious Dose 50% , IM = intramuscular

Blood Sampling schedule, assays and cut-offs for antibodies to vaccine antigens

| **Antibody tested** | **Method** | **Assay cut-off** | **Pre-vaccination**  **(screening: age 8-12 weeks)** | **One month**  **Post-dose 3** | **Two months before the booster at age 18 months** | **One month**  **post-booster** | **24 months post-dose 3 of RTS,S/AS01 or HBV (Month 26)** |
| --- | --- | --- | --- | --- | --- | --- | --- |
| HBs | CLIA | 6.2 mIU/ml | All groups | All groups | All groups |  | All groups |
| CS | ELISA | 0.5/EU/ml | All groups | All groups | All groups |  | All groups |
| PT, FHA, PRN | ELISA | 5 EU/ml | All groups | All groups |  |  |  |
| HBV RF1 | ELISA | 33 EU/ml |  | All groups |  |  |  |
| Pneumococcal polysaccharides 1, 4, 5, 6B, 7F, 9V, 14, 18C, 19F, 23F | ELISA | 0.05 μg/ml |  | C1, R1 |  | C1, R1 |  |
|  | OPA | 1:8 dilution |  |  |  |  |  |
| Protein D | ELISA | 100 EU/ml |  |  |  |  |  |
| HRV IgA | ELISA | 20 U/ml |  |  |  |  |  |

HBs = hepatitis B surface antibody, CS = circumsporozoite protein, PT = pertussis toxin, FHA = filamentous haemagglutinin, PRN = pertactin, HBV = Hepatitis B virus, HRV = human rotavirus, CLIA = Chemiluminescence enzyme immunoassay (Centaur™, Siemens, Germany) ELISA = enzyme linked immunosorbent assay, OPA = opsonophagocytic activity

**Evaluation of safety and assessment of causality**

All solicited local (injection site) reactions were considered causally related to vaccination. Causality of all other adverse events was assessed by the investigator using the following question:

*Is there a reasonable possibility that the* adverse event *AE may have been caused by the investigational product?*

- NO: The AE was not causally related to administration of the study vaccine(s). There were other, more likely causes and administration of the study vaccine(s) was not suspected to have contributed to the adverse event.
- YES: There was a reasonable possibility that the vaccine(s) contributed to the adverse event.

**Intensity grading for solicited symptoms**

| **Adverse Event** | **Intensity grade** | **Parameter** |
| --- | --- | --- |
| Pain at injection site | 0 | None |
|  | 1 | Mild: Minor reaction to touch |
|  | 2 | Moderate: Cries/protests on touch |
|  | 3 | Severe: Cries when limb is moved/spontaneously painful |
| Redness* and swelling | 0 | None |
| at injection site | 1 | Mild: <5 mm |
|  | 2 | Moderate: 5-20 mm |
|  | 3 | Severe: >20 mm |
| Fever** | 0 | < 37.5°C |
|  | 1 | Mild: ≥37.5°C - ≤38.5°C |
|  | 2 | Moderate: > 38.5°C - ≤39.5°C |
|  | 3 | Severe: > 39.5°C |
| Irritability/Fussiness | 0 | Behaviour as usual |
|  | 1 | Mild: Crying more than usual/ no effect on normal activity |
|  | 2 | Moderate: Crying more than usual/ interferes with normal activity |
|  | 3 | Severe: Crying that cannot be comforted/ prevents normal activity |
| Drowsiness | 0 | Behaviour as usual |
|  | 1 | Mild: Drowsiness easily tolerated |
|  | 2 | Moderate: Drowsiness that interferes with normal activity |
|  | 3 | Severe: Drowsiness that prevents normal activity |
| Loss of appetite | 0 | Appetite as usual |
|  | 1 | Mild: Eating less than usual/ no effect on normal activity |
|  | 2 | Moderate: Eating less than usual/ interferes with normal activity |
|  | 3 | Severe: Not eating at all |

* In case the principal investigator or designate was unable to determine the extent of redness on darkly pigmented skin, it was reported as uninterpretable.

** Fever was defined as: rectal temperature ≥38°C /axillary temperature ≥37.5°C /oral temperature ≥37.5°C /tympanic temperature on oral setting ≥37.5°C, tympanic temperature on rectal setting ≥38°C. The preferred route for recording temperature in this study was axillary.

For each solicited and unsolicited symptom the subject experiences, the subject’s parent(s)/LAR(s) were asked if the subject received medical attention defined as hospitalization, an emergency room visit or a visit to or from medical personnel (medical doctor) for any reason and this information was to be recorded in the case report form

**Supplement Figure 1. Study Design**


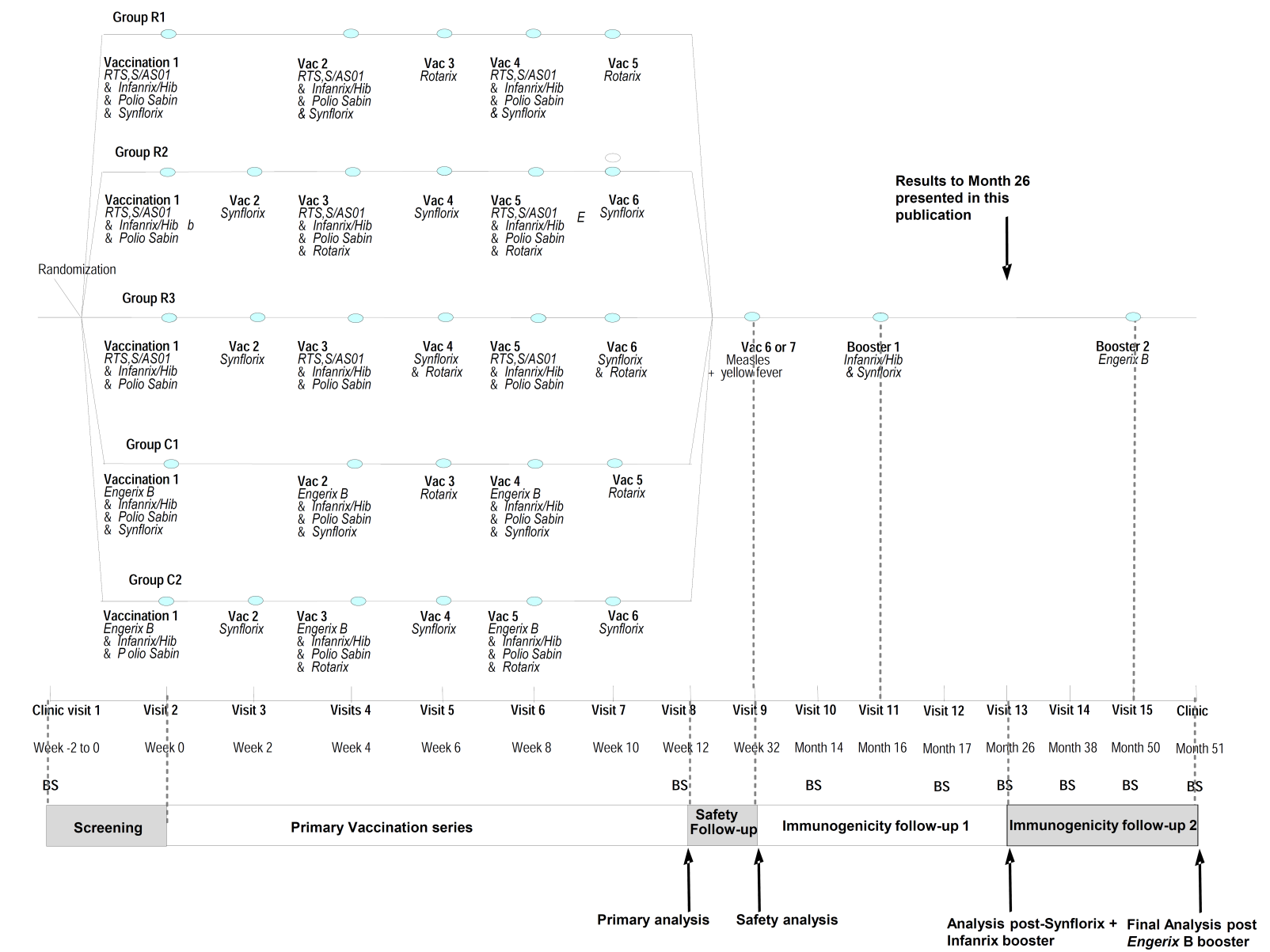


**Supplement figure 2. Fever (axillary temperature ≥37.5°C) recorded daily over 7 days after each dose (Total vaccinated cohort*)**


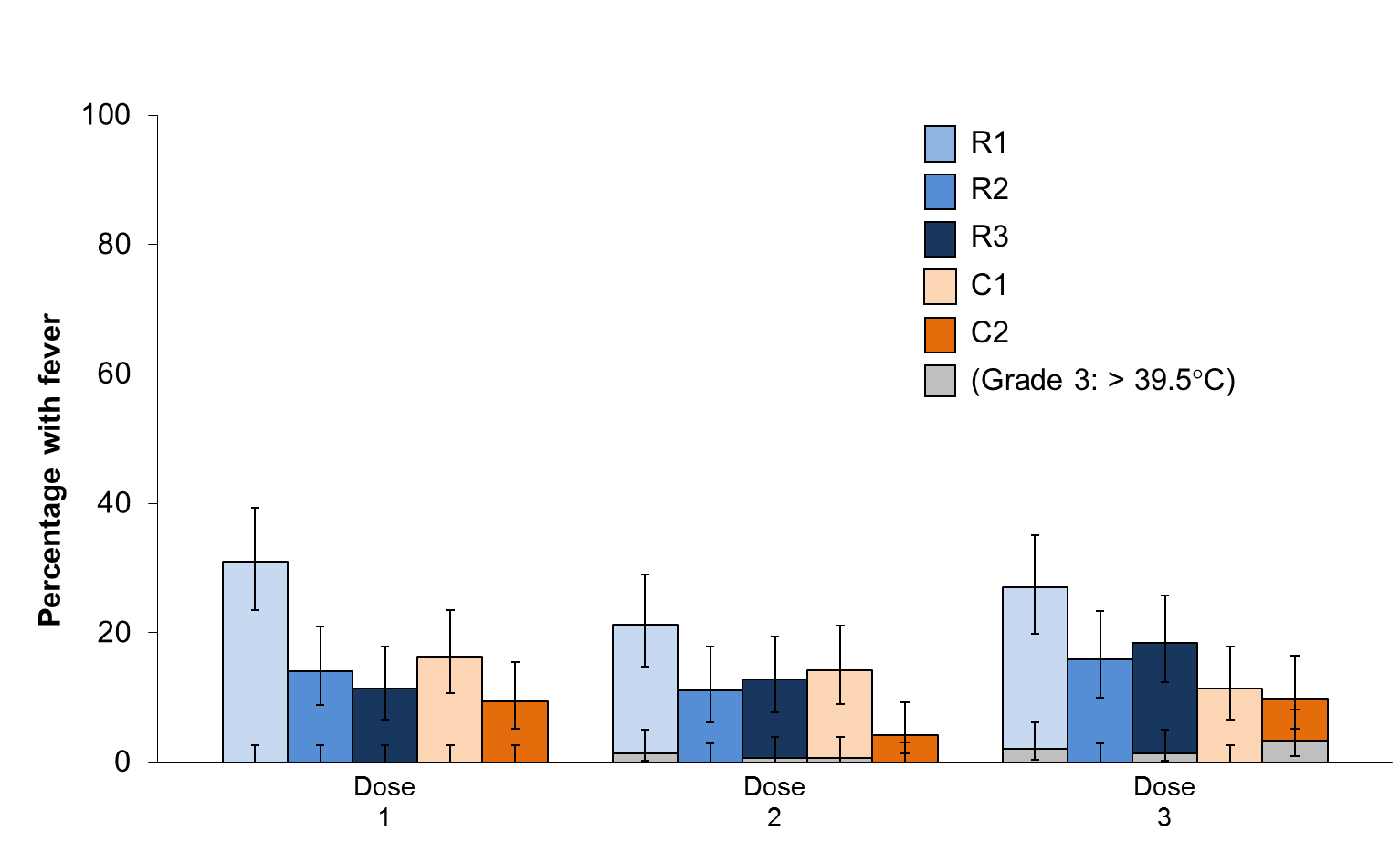


Group R1 received RTS,S/AS01 + (DTPa/Hib + tOPV + PHiD-CV), and HRV 2 weeks later

Group R2 received RTS,S/AS01 + (DTPa/Hib + tOPV + HRV), and PHiD-CV 2 weeks later

Group R3 received RTS,S/AS01 + (DTPa/Hib + tOPV), and (PHiD-CV + HRV) 2 weeks later

Group C1 received HBV + (DTPa/Hib + tOPV + PHiD-CV), and HRV 2 weeks later

Group C2 received HBV + (DTPa/Hib + tOPV + HRV), and PHiD-CV 2 weeks later

% = percentage of doses followed by temperature ≥37.5°C. Grade 3 = temperature > 39.5°C

Vertical lines = Exact 95% confidence interval.

*The analysis of safety was done considering the treatment actually administered
